# The activity and immune dynamics of PD-1 inhibition on high-risk pulmonary ground glass opacity lesions: insights from a single-arm, phase II trial

**DOI:** 10.1038/s41392-024-01799-z

**Published:** 2024-04-19

**Authors:** Bo Cheng, Caichen Li, Jianfu Li, Longlong Gong, Peng Liang, Ying Chen, Shuting Zhan, Shan Xiong, Ran Zhong, Hengrui Liang, Yi Feng, Runchen Wang, Haixuan Wang, Hongbo Zheng, Jun Liu, Chengzhi Zhou, Wenlong Shao, Yuan Qiu, Jiancong Sun, Zhanhong Xie, Zhu Liang, Chenglin Yang, Xiuyu Cai, Chunxia Su, Wei Wang, Jianxing He, Wenhua Liang

**Affiliations:** 1 Department of Thoracic Surgery and Oncology, the First Affiliated Hospital of Guangzhou Medical University, Guangzhou Institute of Respiratory Health, State Key Laboratory of Respiratory Disease, National Clinical Research Center for Respiratory Disease, Guangzhou, China; 2 Medical Department, Genecast Biotechnology Co., Ltd, Wuxi, China; 3 Department of Radiation Oncology, the First Affiliated Hospital of Guangzhou Medical University, Guangzhou, China; 4 Department of Respiratory Medicine, the First Affiliated Hospital of Guangzhou Medical University, Guangzhou Institute of Respiratory Health, State Key Laboratory of Respiratory Disease, National Clinical Research Center for Respiratory Disease, Guangzhou, China; 5 Department of Cardiothoracic Surgery, Affiliated Hospital of Guangdong Medical University, Zhanjiang, China; 6 Department of Thoracic Surgery, National Cancer Center/National Clinical Research Center for Cancer/Cancer Hospital & Shenzhen Hospital, Chinese Academy of Medical Sciences and Peking Union Medical College, Shenzhen, China; 7 Department of VIP Inpatient, Sun Yat-sen University Cancer Center, State Key Laboratory of Oncology in South China, Sun Yat-sen University, Guangzhou, China; 8 Department of Medical Oncology, Shanghai Pulmonary Hospital, School of Medicine, Tongji University, Shanghai, China

**Keywords:** Lung cancer, Tumour immunology

## Abstract

Immune checkpoint inhibitors targeting the programmed cell death-1 (PD-1) protein significantly improve survival in patients with advanced non-small-cell lung cancer (NSCLC), but its impact on early-stage ground-glass opacity (GGO) lesions remains unclear. This is a single-arm, phase II trial (NCT04026841) using Simon’s optimal two-stage design, of which 4 doses of sintilimab (200 mg per 3 weeks) were administrated in 36 enrolled multiple primary lung cancer (MPLC) patients with persistent high-risk (Lung-RADS category 4 or had progressed within 6 months) GGOs. The primary endpoint was objective response rate (ORR). T/B/NK-cell subpopulations, TCR-seq, cytokines, exosomal RNA, and multiplexed immunohistochemistry (mIHC) were monitored and compared between responders and non-responders. Finally, two intent-to-treat (ITT) lesions (pure-GGO or GGO-predominant) showed responses (ORR: 5.6%, 2/36), and no patients had progressive disease (PD). No grade 3–5 TRAEs occurred. The total response rate considering two ITT lesions and three non-intent-to-treat (NITT) lesions (pure-solid or solid-predominant) was 13.9% (5/36). The proportion of CD8^+^ T cells, the ratio of CD8^+^/CD4^+^, and the TCR clonality value were significantly higher in the peripheral blood of responders before treatment and decreased over time. Correspondingly, the mIHC analysis showed more CD8^+^ T cells infiltrated in responders. Besides, responders’ cytokine concentrations of EGF and CTLA-4 increased during treatment. The exosomal expression of fatty acid metabolism and oxidative phosphorylation gene signatures were down-regulated among responders. Collectively, PD-1 inhibitor showed certain activity on high-risk pulmonary GGO lesions without safety concerns. Such effects were associated with specific T-cell re-distribution, EGF/CTLA-4 cytokine compensation, and regulation of metabolism pathways.

## Introduction

Lung cancer is the second most commonly diagnosed cancer and the leading cause of cancer death worldwide, of which approximately 85% are non-small cell lung cancer (NSCLC).^1,2^ The overall survival (OS) of patients with advanced NSCLC was significantly prolonged with immune checkpoint inhibitors (ICIs) targeting the programmed cell death-1 (PD-1) and programmed death-ligand 1 (PD-L1) axis.^3–5^ For early-stage lung cancer, the 5-year survival rate for patients ranges from 80% in stage IA to 41% in stage IIIA, and many cases relapse after surgical resection.^6^ Currently, multiple clinical trials have manifested the encouraging efficacy of neoadjuvant immunotherapy in stage I-IIIA resectable NSCLC.^7–9^ However, the effect of immunotherapy in ultra early-stage NSCLC patients with micro-invasive or even pre-invasive lesions remains unclear.

With the implementation of computed tomography (CT)-guided lung cancer screening, there has been a gradual increase in the detection of pulmonary nodules.^10^ They are classified as solid or sub-solid, with the latter further divided into pure ground-glass opacity (GGO) and part-solid, based on CT appearance.^11^ There is remarkable difference in biological behavior between lung cancers manifesting as different radiological types. Compared with lung cancers presenting with solid nodules, GGO-associated lung cancers have an indolent clinical course, and are characterized by a less active metabolism and a less active immune microenvironment.^12,13^

In recent years, an increasing number of multifocal lung cancers have been diagnosed.^14,15^ Multiple primary lung cancer (MPLC) often presents as multiple GGOs in CT, most of which are minimally invasive or pre-invasive lesions.^16–19^ One routine option is to resect the major lesion(s), followed by close surveillance of the remaining lesions.^20,21^ Generally, it was extremely difficult to remove all lesions for MPLC patients, considering their pulmonary function, comorbidities, and multiple lesions in different lobes, etc. As a clinical dilemma, there is no consensus on the management of unresected lesions with a high risk of progression for MPLC patients after primary surgery.^17,22–25^ Study has revealed that immune escape occurs in the pre-invasive stages of carcinogenesis in the lung.^26^ Therefore, it is promising to investigate the utility of PD-1 inhibitors on high-risk pulmonary GGO lesions.

Exploring the immune dynamics during PD-1 blockade treatment in such early diseases is of great interest. T cell responses are critical for anti-tumor immunity in cancer patients, and the state of circulating and tumor-infiltrating T cells is associated with patients’ responses to immunotherapy.^27^ In addition, previous studies demonstrated that the characteristics (clonality and diversity) of T-cell receptor (TCR) might be potential biomarkers for NSCLC patients treated with ICIs.^28,29^

We herein conducted this single-arm, phase II trial (CCTC-1901, NCT04026841) with Simon’s two-stage design, aiming to evaluate the activity and safety of sintilimab on high-risk GGO lesions in MPLC patients.

## Results

**Fig. 1:**
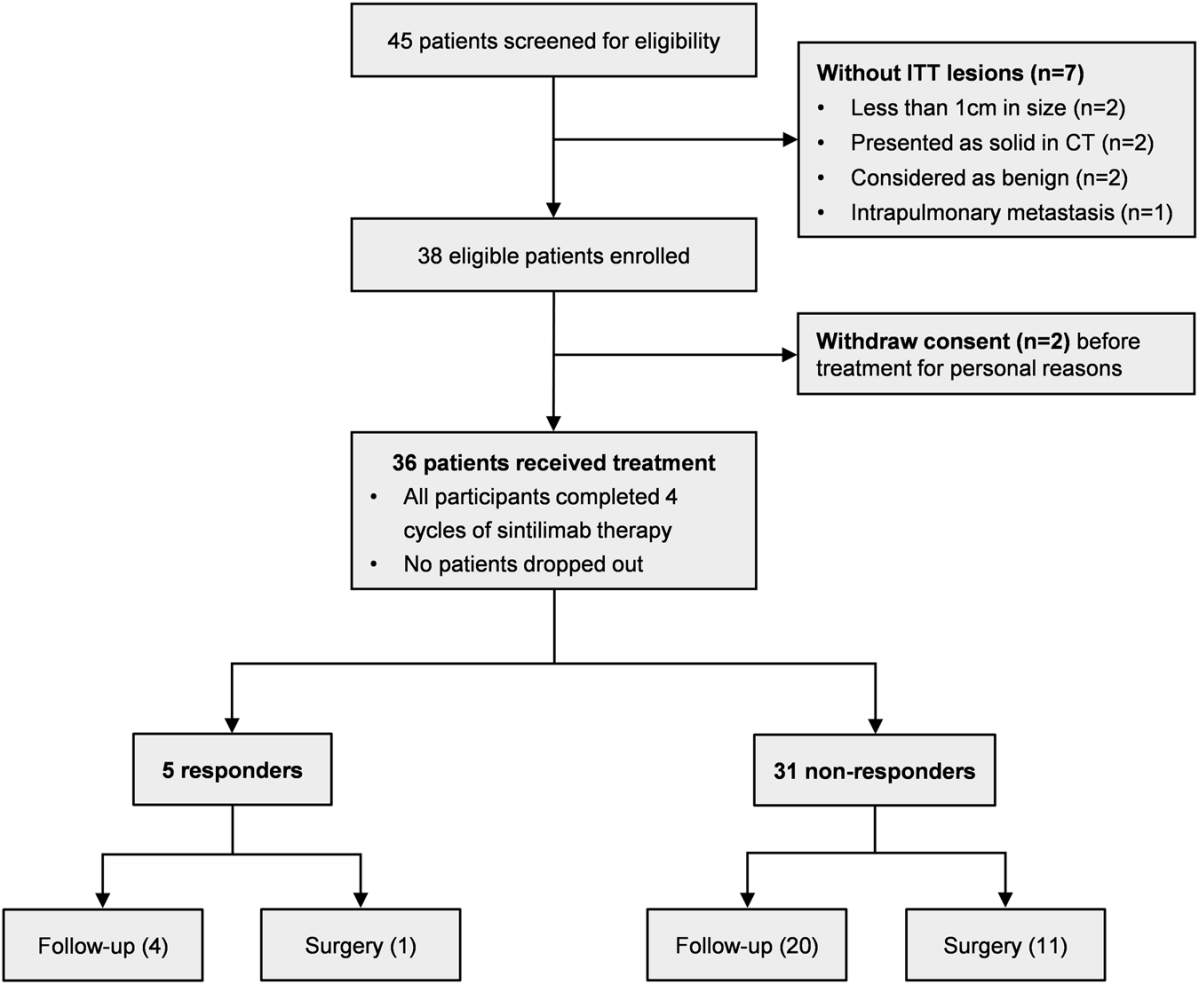
Flow diagram of screening eligible patients for inclusion. The flow chart depicts the reasons for screening failures, and a total of 36 patients were included in this trial

**Fig. 2:**
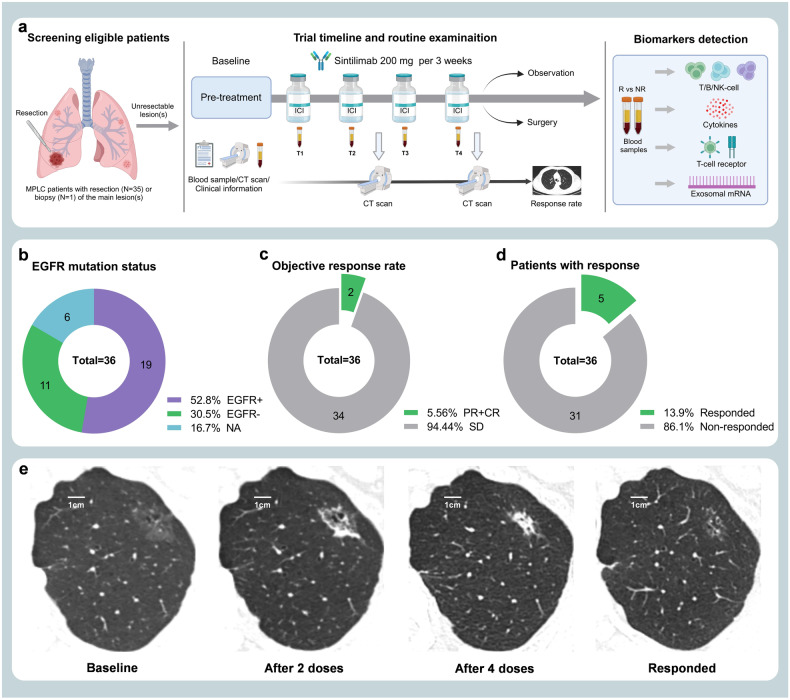
Trial timeline, routine examinations, and outcome of the study. **a** After baseline information collection, enrolled patients received intravenous drip sintilimab 200 mg per 3 weeks for 4 cycles; blood sampling before every cycle (labeled as T1–T4) and chest CT scan after every 2 cycles. Biomarkers including T/B/NK-cell subpopulations, T-cell receptor (TCR), cytokines, and exosomal RNA, were monitored and compared between responders and non-responders. **b** The EGFR mutation status of resected or bioptic main lesion(s) of patients. **c** The objective response rate (ORR: 5.6%, 2/36) based on ITT lesions of patients. **d** The total response rate (13.9%, 5/36) considering two ITT lesions (pure-GGO or GGO-predominant) and three NITT lesions (pure-solid or solid-predominant). **e** The radiologic changes in chest CT of one patient who achieved significant response after sintilimab treatment. (A 78-years-old man with a smoking history; he has one ITT lesion manifesting as mixed GGO and with no NITT lesion)

This is a single-arm, phase II trial using Simon’s optimal two-stage design, of which 4 doses of sintilimab (200 mg per 3 weeks) were administrated in 36 MPLC patients with persistent high-risk GGOs, and an array of translational studies including T/B/NK-cell subpopulations, TCR-seq, Cytokines, Exosomal RNA, and mIHC detections were performed. The flow chart of this study is shown in Fig. 1, and the study design is illustrated as Fig. 2a. The specific details of this study containing the inclusion/exclusion criteria, primary/secondary endpoints, treatment information, and study design were described detailly in the Materials and methods section.

### Characteristics of the patients and ITT lesions

From July 2019 through September 2020, 36 patients were eligible for inclusion in this study (Table 1), with a median age of 59.5 (IQR, 53.5–69). Among them, 66.7% were females, 19.4% were current or former smokers; all their major lesion(s) with resection or biopsy were adenocarcinomas, of them 88.9% were stage I (or earlier), 27.8% were adenocarcinoma in situ (AIS) or minimally invasive adenocarcinoma (MIA), and 52.8% were EGFR mutation-positive (Fig. 2b). Finally, a total of 49 unresected GGOs (pure 11[22.4%], mixed 38[77.6%]) were set as ITT lesions, with a mean size of 13.20 ± 5.06 mm. For the nature and EGFR mutation status of ITT lesions, the prediction results of AI software based on patients’ baseline CT are shown in Supplementary Fig. 1. There were 40 ITT lesions predictable (probability of malignancy ≥0.3) for EGFR mutation status by the AI software, and 47.5% (19/40) of them were predicted as EGFR mutation-positive.

**Table 1:** Patient demographic and baseline characteristics

Characteristic	Patients, NO. (%)
No. of patients	36
Median age, years (IQR)	59.5 (53.5–69)
Sex	
Male	12 (33.3)
Female	24 (66.7)
Smoking history	
Yes	7 (19.4)
No	29 (80.6)
ITT lesions number	
1	27 (75)
2	6 (16.7)
3	2 (5.6)
4	1 (2.8)
Main Lesions	
Diagnosis method	
Biopsy	1 (2.8)
Surgery	35 (97.2)
Lobectomy	17 (47.2)
Segmentectomy	7 (19.4)
Wedge resection	11 (30.6)
Lymph node dissection^a^	
Yes	26 (74.3)
No	9 (25.7)
Pathology^b^	
AIS	1 (2.8)
MIA	9 (25)
IA	26 (72.2)
Time interval, months^c^	7 (1–25)
≤6	17 (48.6)
6–24	8 (22.8)
>24	10 (28.6)
TNM stage	
Tis	1 (2.8)
I	31 (86.1)
II	2 (5.6)
III	2 (5.6)
EGFR mutation	
Positive	19 (52.8)
Negative	11 (30.5)
Not obtained	6 (16.7)
ITT Lesions	
No. of ITT lesions	49
Mean size, mm	13.20 ± 5.06
GGO type	
Pure	11 (22.4)
Mixed	38 (77.6)
NITT Lesions	
No. of NITT lesions	5
Mean size, mm	19.8 ± 6.4
Radiological type	
Pure-solid	3 (60.0)
Solid-predominant	2 (40.0)

^a^Statistics for lymph node dissection were based on 35 patients who underwent surgical resection of the main lesion(s)

^b^*AIS* Adenocarcinoma in situ, *MIA* Minimally invasive adenocarcinoma, *IA* Invasive adenocarcinoma

^c^The time interval (median, IQR) between 35 patients’ primary surgery on the main lesion(s) and the start of sintlimab treatment

### Efficacy and safety

The ORR was 5.6% (2/36); remission was achieved in 2 ITT GGOs (2/49), including 1 complete response (CR) and 1 partial response (PR) (Fig. 2c). Moreover, 3 NITT lesions (unresected solid or solid-predominant lesions) from 3 enrolled patients showed PR after the treatment of sintilimab (total response rate: 13.9%, 5/36), and the rest 31 patients’ lesions showed stable disease (SD) (Fig. 2d). No patients had progressive disease (PD) during the medication. Figure 2e shows the radiologic changes after treatment with sintilimab in one patient achieving a significant remission.

TRAEs of any grade occurred in 26 (72%) of 36 patients, with grade 1 in 17 (47%) patients and grade 2 in 9 (25%) patients, and no grade 3–5 TRAEs were observed throughout the follow-up (Table 2). Fatigue (36%, 13/36) and rash (36%, 13/36) were the most common adverse events. No patient withdrew from the trial due to adverse events.

**Table 2:** Treatment-related adverse events (*N* = 36)

Adverse event^a^	Any Grade	Grade 1	Grade 2
All	26 (72%)	25 (69%)	9 (25%)
Fatigue	13 (36%)	12 (33%)	1 (3%)
Rash	13 (36%)	13 (36%)	0
Arthralgia	8 (22%)	3 (8%)	5 (14%)
Myalgia	6 (17%)	4 (11%)	2 (6%)
Decreased appetite	5 (14%)	5 (14%)	0
Hypothyroidism	5 (14%)	0	5 (14%)
Pruritus	3 (8%)	3 (8%)	0
Dysgeusia	2 (6%)	2 (6%)	0
Insomnia	2 (6%)	2 (6%)	0
Xerostomia	2 (6%)	2 (6%)	0
Nausea	1 (3%)	1 (3%)	0
Cough	1 (3%)	0	1 (3%)
Diarrhea	1 (3%)	1 (3%)	0
Dry skin	1 (3%)	1 (3%)	0
Stomatitis	1 (3%)	0	1 (3%)
Hyperthyroidism	1 (3%)	1 (3%)	0
Neutropenia	1 (3%)	1 (3%)	0
White blood cell count decreased	1 (3%)	1 (3%)	0

Data are *n* (%). The table shows treatment-related adverse events that occurred in any enrolled patients. Adverse events were graded according to the National Cancer Institute Common Terminology Criteria for Adverse Events, version 5.0

^a^No grade 3–5 adverse events occurred in all 36 included patients

### Patient outcome after treatment

When the full medication is completed, the investigators will make subsequent treatment strategies for patients by comprehensively considering their tolerance to surgery, their personal willingness (conservative or surgical treatment), and the invasiveness (nodule size, solid components, etc.) of the lesions. Of 5 patients who showed a response, 1 underwent surgery under the patient’s request (for a NITT lesion achieving PR, and an ITT lesion without remission), and 4 received follow-up observation; for 31 patients without response, 11 underwent surgery, and 20 received follow-up observation. Surgical information of the second surgery after sintimab treatment in enrolled patients is provided in Supplementary Table 1. No patient was observed to have progression throughout the follow-up after treatment.

A total of 13 ITT lesions from 12 patients were surgically resected, including 3 cases of IA and 10 cases of MIA. Gene testing was performed on the tumor specimens from 9 patients, and all of them (9/9) were EGFR mutation-positive. Notably, two ITT lesions that showed responses to sintilimab treatment were predicted by the AI software since they were not resected, and no tissue was obtained for the gene test. Among them, one was predicted as EGFR mutation-positive, and one was EGFR mutation-negative, indicating that EGFR-mutated GGO lesions might be able to benefit from immunotherapy.

### mIHC

**Fig. 3:**
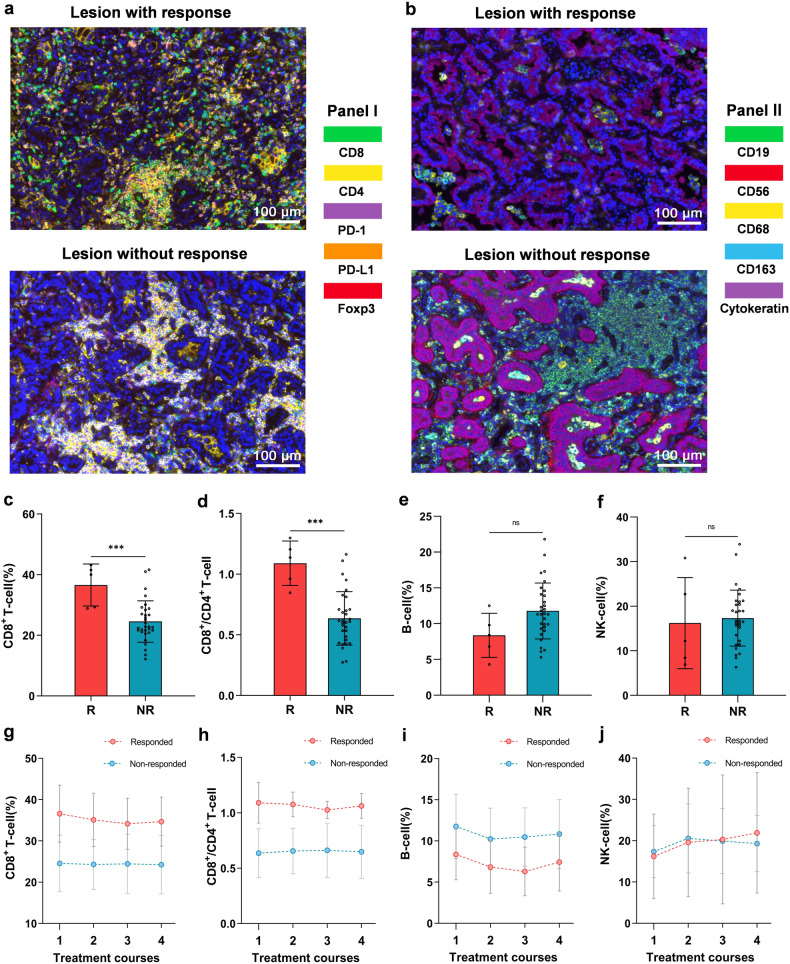
The mIHC analysis and T/B/NK-cell detection of responders and non-responders. **a**, **b** The mIHC results of 1 responded and 1 non-responded tumor. These structures could be visible by staining, including panel I (**a**): CD8^+^ T cells (green), CD4^+^ T cells (yellow), PD-1^+^ cells (magenta), PD-L1^+^ cells (orange), Foxp3^+^ regulatory T cells (red); and panel II (**b**): CD19^+^ B cells (green), CD56^+^ NK cells (red), CD68^+^ macrophages (yellow), CD163^+^ M2 macrophages (cyan), cytokeratin^+^ tumor cells (magenta). The proportion of CD8^+^ T cells (**c**), the ratio of CD8^+^/CD4^+^ T-cell (**d**), and the proportion of B cells (**e**) and NK cells (**f**) in the pre-treatment blood (T1) of responders (red, *n* = 5) and non-responders (blue, *n* = 31). The changing trend of immune cells over time (T1–T4) in responders (red, *n* = 5) and non-responders (blue, *n* = 31), including the proportion of CD8^+^ T cells (**g**), the ratio of CD8^+^/CD4^+^ T-cell (**h**), and the proportion of B cells (**i**) and NK cells (**j**). Data (**c**–**f**) are shown as means ± SD, with *P < 0.05, **P < 0.01, ***P < 0.001 and ‘ns’ for no significant difference

The mIHC analysis was implemented based on 1 responded (only 1 patient underwent surgery in responders) and 5 non-responded tumor issues. These structures could be visible by staining, including panel I: CD8^+^ T cells, CD4^+^ T cells, PD-1^+^ cells, PD-L1^+^ cells, Foxp3^+^ regulatory T cells (Fig. 3a); and panel II: CD19^+^ B cells, CD56^+^ NK cells, CD68^+^ macrophages, CD163^+^ M2 macrophages, cytokeratin^+^ tumor cells (Fig. 3b). Compared with the non-responded tumors, it seemed that the responded tumor was infiltrated with more CD8^+^ T cells, less CD4^+^ T cells, less CD19^+^ B cells, and less CD163^+^ M2 macrophages. The detailed proportions of various immune cells in mIHC are shown in Supplementary Table 2. Figure 3a, b shows the staining of 1 responded and 1 non-responded tumor, and mIHC results of all patients are available in Supplementary Fig. 2a, b. Clinical course and timing of surgery of the patients receiving mIHC detection are illustrated in Supplementary Fig. 3.

### T/B/NK-cell subpopulations

Blood samples of all included patients were collected before every administration, and labeled as T1–T4 according to the cycles of sintilimab treatment. For the T/B/NK-cell detection in baseline blood (T1), the proportion of CD8^+^ T cells and the ratio of CD8^+^/CD4^+^ T-cell in 5 responded patients were significantly higher than those (31 patients) without response (Mean: CD8^+^ 36.6% vs. 24.6%, *p* < 0.001; CD8^+^/CD4^+^ 1.09 vs. 0.64, *p* < 0.001) (Fig. 3c, d). In addition, the proportion of B cells in responded patients was lower than that in non-responded patients (Mean: 8.4% vs. 11.8%, *p* = 0.0723), and the proportion of NK cells was similar in these two groups (Mean: 16.2% vs. 17.3%, *p* = 0.7345), with no significant difference (Fig. 3e, f). The absolute counting of CD8^+^ and CD4^+^ T cells is shown in Supplementary Fig. 4a, b.

The changing trend of the proportion of various immune cells over medication cycles in responders and non-responders was monitored. The proportion of CD8^+^ T cells in responders decreased gradually from T1 to T3, while the non-responders showed an almost unchanged trend (F = 2.210, *p* = 0.118, partial η^2^ = 0.061), which was coincident with the variation trend of the ratio of CD8^+^/CD4^+^ T-cell of patients in these two groups (F = 1.288, *p* = 0.282, partial η^2^ = 0.037), but with no statistical difference (Fig. 3g, h). Besides, the variation trend of the proportion of B cells (F = 0.214, *p* = 0.828, partial η^2^ = 0.006), as well as of NK cells (F = 1.308, *p* = 0.276, partial η^2^ = 0.037) were similar throughout T1–T4 in patients of both groups (Fig. 3i–j). The changes of the absolute counting of CD8^+^ and CD4^+^ T cells from T1 to T4 are illustrated in Supplementary Fig. 4c, d. The changes of immune cells of each enrolled patient (*N* = 36) are shown in Supplementary Fig. 5a–f.

### TCR-seq, cytokines and exosomal RNA

Examinations including TCR-seq, cytokines, and exosomal RNA were conducted using the blood samples from 5 responded patients (group-R) and 5 representative non-responded patients (group-NR) matched by similar clinical characteristics. The baseline information of these 10 patients is provided in Supplementary Table 3.

**Fig. 4:**
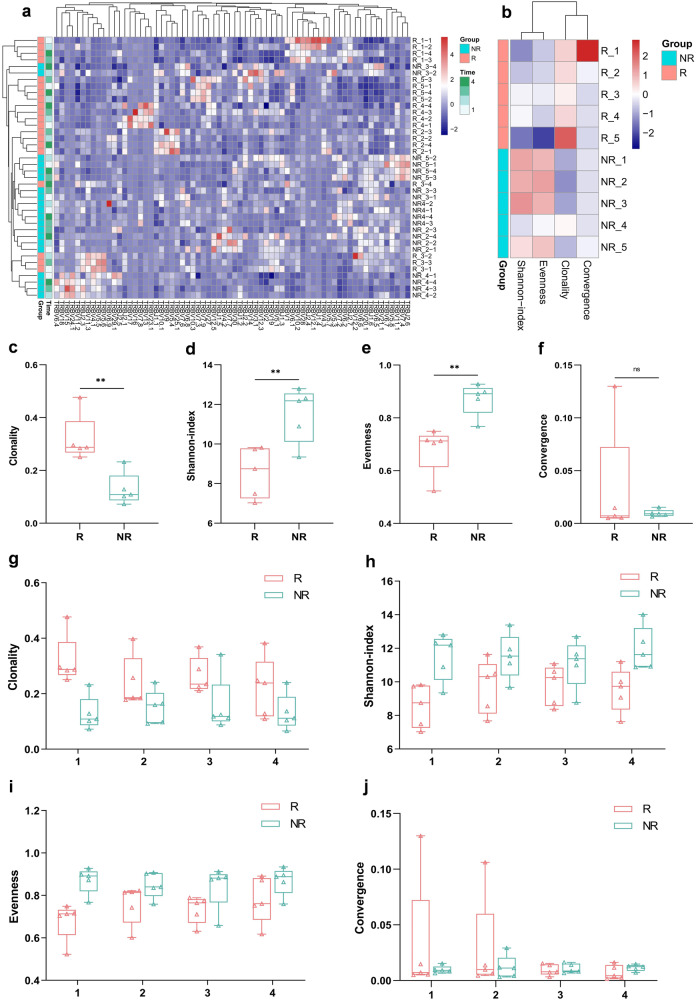
The TCR features of group-R (*n* = 5) and group-NR (*n* = 5) patients. **a** The clustering analysis was performed by patients’ features of TCR throughout the treatment (T1–T4). Patients from the same group were clustered, and group-R and -NR exhibited significantly distinct patterns of TCR repertoire. The comparison of TCR features (**b**) between patients in group-R (red) and -NR (green) based on pre-treatment blood (T1), including the value of TCR clonality (**c**), Shannon-index (**d**), evenness (**e**), and convergence (**f**). The variation tendency of TCR clonality (**g**), Shannon-index (**h**), evenness (**i**), and convergence (**j**) of two-group patients after medication (T1–T4). Data (**c**–**j**) are shown as median (min to max), with *P < 0.05, **P < 0.01, ***P < 0.001 and ‘ns’ for no significant difference

Clustering analysis was performed for patients by their features of TCR from T1–T4. As shown in Fig. 4a, the horizontal axis showed different TCR types, and the vertical axis showed the test results of patients (5 responders [R1-R5] and 5 non-responders [NR1-NR5]) at different time points (T1/T2/T3/T4). Patients from the same group were clustered, and group-R and -NR exhibited significantly distinct patterns of TCR repertoire. Meanwhile, the TCR clonality and diversity including Shannon-index, evenness, and convergence were analyzed based on patients’ pre-treatment blood samples (T1) (Fig. 4b). In comparison to the patients in group-NR, the value of baseline TCR clonality was significantly higher (Mean: 0.319 vs. 0.129, *p* < 0.01), and the Shannon-index and evenness were significantly lower in group-R (Mean: 8.565 vs. 11.504, *p* < 0.01; 0.681 vs. 0.872, *p* < 0.01, respectively); there was no significant difference in convergence between two groups (Mean: 0.032 of group-R vs. 0.01 of group-NR, *p* = 0.407) (Fig. 4c–f). The variation of these indicators after medication (T1 to T4) was also monitored. The clonality value showed a downtrend, and the Shannon-index and evenness value showed an uptrend in responders, but no remarkable changes of these indicators appeared in non-responders (Fig. 4g–i); there was no obvious variation of convergence in both two-group patients (Fig. 4j).

**Fig. 5:**
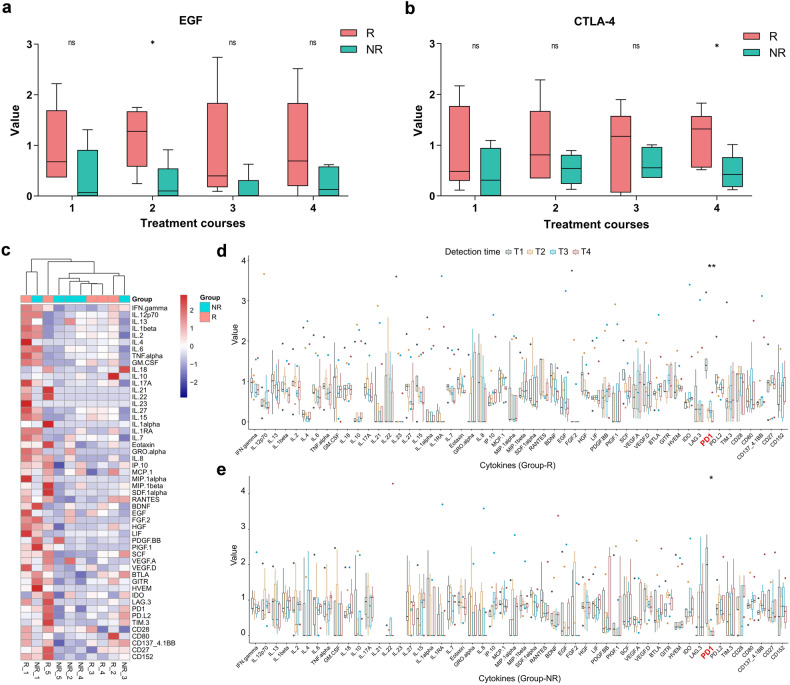
The results of cytokines detection of group-R (*n* = 5) and group-NR (*n* = 5) patients. The concentration values of EGF (**a**) and CTLA-4 (**b**) of patients in group-R (red) and group-NR (green) at every time point (T1–T4). **c** The clustering of two-group patients based on cytokines in the pre-treatment blood (T1). The concentration values of various cytokines at different time points (T1–T4) in group-R (**d**) and group-NR (**e**) patients. With *P < 0.05, **P < 0.01, ***P < 0.001 and ‘ns’ for no significant difference

An examination including 45 cytokines and 14 immune checkpoints was performed, and the corresponding concentration values were then standardized using the Z-score (to a mean of zero and standard deviation of 1). There was no significant difference in various cytokines between two-group patients at every time point (T1–T4) (Supplementary Fig. 6a–d), except higher EGF at T2 (*p* = 0.027) and higher CTLA-4 (CD152) at T4 (*p* = 0.046) in group-R patients (Fig. 5a, b). Figure 5c illustrates the clustering of patients based on cytokines in baseline blood (T1), showing no discrimination between the two groups. What’s more, we also compared the cytokines concentration of patients at different courses (T1–T4), and no significant changes appeared for all cytokines except that the level of PD-1 was dramatically decreased in both group-R (*p* < 0.01) and -NR (*p* = 0.028) after administration of sintilimab (Fig. 5d, e).

**Fig. 6:**
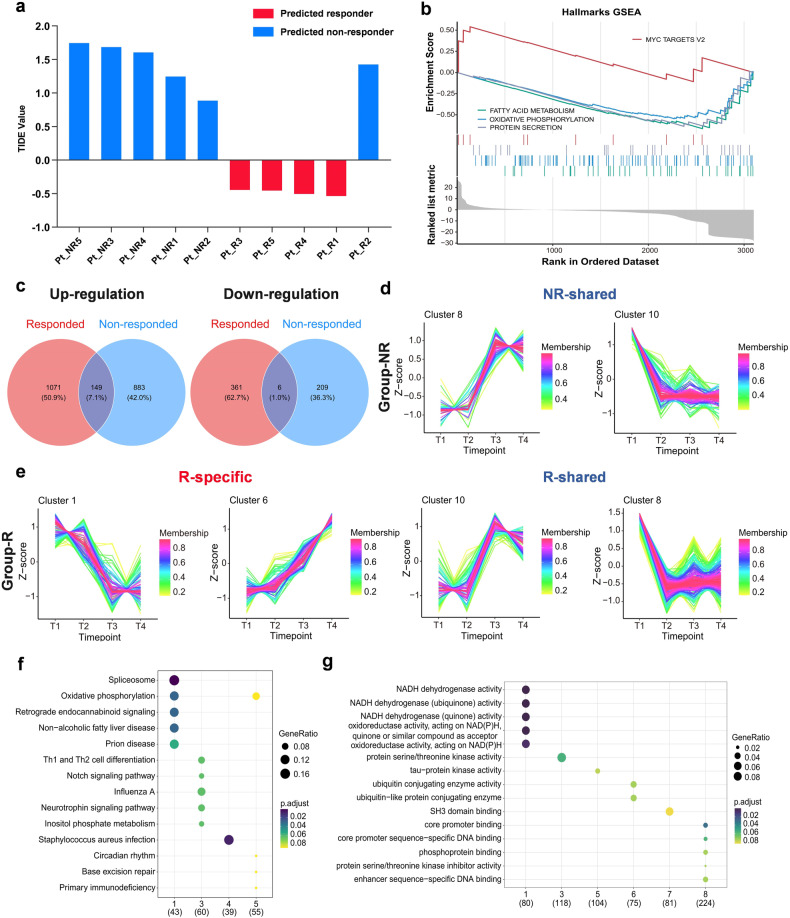
The gene expression profiles of group-R (*n* = 5) and group-NR (*n* = 5) patients. **a** The availability of the obtained exosomal RNA of patients was verified using the “TIDE” model, and patients were predicted as responders (red) or non-responders (blue). **b** Gene set enrichment analysis (GSEA) according to “Hallmark” gene sets from MsigDB. **c** Up- and down-regulated genes after medication (T2/T3/T4 vs. T1) in group-R (red) and group-NR (blue) patients. Time-series clustering analysis was taken to observe the trend of genes expression over time (T1–T4) in patients of group-NR (**d**) and -R (**e**); the horizontal axis showed different time points, and the vertical axis showed the gene expression after standardization, which was the mean expression value of patients in each group for one certain gene. KEGG (**f**) and GO (**g**) enrichment analyses according to different time-series clusters

We analyzed the obtained exosomal RNA of patients using “TIDE”,^30^ which was a computational method to accurately predict the ICIs clinical response based on pre-treatment tumor expression profiles, and 9 out of 10 patients were eventually correctly predicted (4 from group-R and 5 from group-NR) (Fig. 6a). Then the gene set enrichment analysis (GSEA) was performed to compare the gene expression profiles between group-R and -NR patients, according to all 50 “Hallmark” gene sets from MsigDB (Fig. 6b).^31^ For patients in group-R (vs. -NR), the up-regulated gene signature was Myc targets_v2, and the down-regulated gene signatures included Fatty acid metabolism, Oxidative phosphorylation (OXPHOS), and Protein secretion. Besides, up- and down-regulated genes after administration (T2/T3/T4 vs. T1) were assessed separately within two groups. As illustrated in Fig. 6c, for responded and non-responded patients, 1220 and 1032 genes were up-regulated, and 367 and 215 genes were down-regulated, respectively; either up- or down-regulated genes showed only a few overlaps between the two groups (149 [7.1%] and 6 [1.0%], respectively).

A time-series clustering analysis was taken to observe the gene expression trends over time (T1 to T4) in responders and non-responders, and the genes with similar expression trends would be gathered in one same cluster. Finally, 10 clusters (time-series gene expression trends) were identified in group-R and group-NR respectively, and each line represented one gene (Supplementary Figs. 7 and 8). Thereinto, 4 clusters in group-R and 2 clusters in group-NR displayed remarkable up- or down-regulation of gene expression after medication (Fig. 6d, e). Notably, in group-R, the trends represented by cluster 1 and 6 were specific, and the other two clusters (cluster 8 and 10) shared the same trend with group-NR. We proceeded with the KEGG (Kyoto Encyclopedia of Genes and Genomes) and Gene Ontology (GO) enrichment analyses, to reveal these genes and pathways represented by two specific clusters (Fig. 6f, g). In group-R, cluster 1 mainly presented the down-regulation of oxidative phosphorylation and non-alcoholic fatty liver disease pathways, and down-regulated genes expression related to NADH dehydrogenase activity and oxidoreductase activity; and cluster 6 presented the up-regulation of genes expression of ubiquitin conjugating enzyme activity.

## Discussion

CCTC-1901 is the first reported, single-arm, phase II prospective trial testing sintilimab on high-risk GGO lesions in patients with MPLC, using ORR as the primary endpoint. Sintilimab showed certain activity on these early-stage lesions, with 5.6% (2/36, ITT lesions) and 13.9% (5/36, ITT and NITT lesions) of patients achieving radiologic responses. The toxicity profile was manageable without grade 3–5 AEs or new safety concerns. Furthermore, the immune dynamics of patients treated with PD-1 inhibitor in such early diseases were closely monitored in this study, which has not been reported before. This novel strategy showed a favorable potential of PD-1 inhibitor for treating MPLC patients, and it might take an important role in the medication of early-stage lung cancer.

Given the small size and hard-to-access location of these high-risk GGO lesions (persistent for ≥1 year; Lung-RADS category 4 or had progressed within 6 months), the tissue biopsy was infeasible as an invasive approach. Therefore, we adopted the MDT diagnosis, a method being admitted in clinical practice,^32^ to assess the nature of GGOs. To explore the immune dynamics of PD-1 inhibition in patients with high-risk pulmonary GGO lesions, we scrutinized and monitored various blood-based biomarkers, encompassing T/B/NK-cell subpopulations, TCR, cytokines, and exosomal RNA, with a comparative focus on responders and non-responders. Moreover, the mIHC analysis was performed to observe tumor-infiltrating immune cells.

In the mIHC analysis, compared to non-responded tumor, it seemed that responded tumor was infiltrated with more CD8^+^ T cells, and less CD4^+^ T cells, CD19^+^ B cells and CD163^+^ M2 macrophages. This was consistent with previous findings that the presence of high CD8^+^ tumor-infiltrating lymphocytes (TILs) was a biomarker for better efficacy of immunotherapy in cancer patients^33,34^, and the M2 macrophages and certain B-cell subsets in the tumor microenvironment could suppress anticancer immunity and promote tumor growth.^35–38^ In addition, for biomarkers in the pre-treatment peripheral blood, Cheng et al. found that the ratio of CD4^+^/CD8^+^ and the frequency of CD4^+^ T cells might be crucial independent biomarkers to for anti-PD-1 immunotherapy,^39^ and Duchemann et al. reported that the CD8^+^PD-1^+^ to CD4^+^PD-1^+^ ratio was associated with the clinical benefit of NSCLC patients treated with ICIs.^40^ Likewise, in the present study, we observed that the proportion of CD8^+^ T cells and the ratio of CD8^+^/CD4^+^ in the baseline blood of responders were significantly higher compared with non-responders, and the proportion of B cells was lower in responders. In responded patients, the gradual decrease in the proportion of CD8^+^ T cells observed in peripheral-blood after treatment might be attributed to their increased migration into the tumor tissue.^41,42^ Remarkably, the radiologic changes exhibited by a responder in Fig. 2e were consistent with this phenomenon.

There have been studies suggesting that cancer patients with higher TCR clonality had improved clinical responses to ICIs.^9,28,43^ Additionally, several investigations demonstrated that higher TCR diversity was associated with superior response to anti-PD-1/PD-L1 therapy,^29,42,44^ whereas Zhang et al. reported a converse finding that patient with lower TCR diversity achieved better benefit from immunotherapy.^45^ In this study, the features of peripheral blood-based TCR repertoire significantly differed in patients with and without response. Prior to treatment, responders exhibited higher TCR clonality and lower diversity (Shannon-index and evenness) compared to that in non-responders. Following the administration of sintilimab, responders displayed a declining trend in clonality value, along with an ascending trend in Shannon-index and evenness values. These results were in concord with the dynamic changes of CD8^+^ T cells we observed, in which a group of dominant clones accounted for higher clonality initially, resulting in the lower TCR diversity; whereafter, more ICIs-induced migration of T cells into the tumor led to an increase of TCR diversity in the blood.^8,46^

A total of 45 cytokines and 14 immune checkpoints in the peripheral blood were tested in our study. Only EGF at T2 and CTLA-4 at T4 presented significantly higher values in responded patients, and no statistical difference was found in the rest of the cytokines between two-group patients throughout T1 to T4. This might reflect the immune escape of tumor and CTLA4-mediated regulation of immunity in the responders. Studies revealed that cancer cells could secret EGF to activate the EGFR signaling pathway and suppress antitumor immunity.^47,48^ After PD-1-targeted therapies, a high level of CTLA-4 was expressed in the peripheral blood to regulate T-cell activity.^49,50^

To further compare the gene expression profiles between patients with different responses, the gene set enrichment analysis (GSEA) was employed. The Myc targets_v2 gene signature was significantly enriched in group-R patients, and the gene signatures of Fatty acid metabolism and OXPHOS were markedly enriched in group-NR patients, which corresponded to our results of KEGG and GO enrichment analyses. These findings were supported by previous studies, the up-regulation of Fatty acid metabolism and OXPHOS gene signatures were associated with treatment failure of ICIs.^51,52^ Additionally, the enrichment of gene signature of Myc targets_v2 in responders might be owing to the MYC-driven immune evasion and the activation of EGFR signaling pathway.^53–55^

In previous retrospective observational studies, Wu et al. proposed that the GGO lesions might achieve benefit from immunotherapy in patients with advanced lung adenocarcinoma, and Zhang et al. reported a case that one solid nodule of an MPLC patient showed significant shrinkage after ICI treatment.^45,56^ Xu et al. carried out a phase I clinical trial and found that the toxicity of using ICI in MPLC patients was controllable.^57^ Previously, the PEARLS/KEYNOTE-091 study provided evidence supporting pembrolizumab as adjuvant therapy in resected stage IB–IIIA NSCLC. However, all patients included in that study (stage IB patients accounted for 14%) were with invasive tumors.^58^ In our study, most of the enrolled patients had the disease in stage IA or earlier (MIA; AIS). Therefore, whether the immunotherapy showed activity in the very early-stage (even pre-invasive) lung cancer could be observed in this trial.

In this prospective phase II intervention study, we confirmed that the PD-1 inhibitor was active for high-risk pulmonary GGO lesions. Besides, none of the enrolled patients progressed and no emergence of new GGO lesions occurred during the treatment and subsequent follow-up. This might be attributed to the reasons that: PD-1 blockade has a favorable effect of disease control on these high-risk GGOs and shows prophylactic potency against the development of new GGOs; or many GGOs are naturally indolent and grow slowly. It warrants validation of whether the PD-1 inhibitor has a long-term effect on these lesions. Thus, PD-1 inhibition might enhance the immune surveillance in patients and prevent the formation of tumors at a very early stage, which needs to be further confirmed by prospective studies.

Our study is the first to evaluate the activity and safety of sintilimab on high-risk GGO lesions in MPLC patients, and there has been no relevant research before. In this study, we did not exclude the patients who harbored EGFR mutation (detected on the main lesion or predicted by artificial intelligence), aiming to explore the efficacy of immunotherapy on high-risk lesions in MPLC patients with or without EGFR alteration. In this regard, we mainly have the following considerations: Firstly, in MPLC patients, different lesions are independent of each other, with significant genetic heterogeneity, and different lung cancer lesions in the same individual may have a high discrepancy of driver mutations, which is led by distinct molecular events. Therefore, there was no explicit association between the gene mutation status among multiple primary lesions;^59,60^ Next, previous studies indeed indicated that NSCLC patients with EGFR mutation-positive benefit less from immunotherapy compared to those without EGFR alteration.^61,62^ Nonetheless, the subgroup analyses of PEARLS/KEYNOTE-091 and LCMC3 study, as well as some retrospective studies, revealed that immunotherapy is not completely ineffective in EGFR-mutant NSCLC patients.^58,63,64^ Notably, for this study, in the 2 ITT lesions that showed remission after sintilimab treatment, 1 of them was predicted as EGFR mutation-positive by the AI software. In truth, the immune microenvironment is distinct between the advanced and early-stage lung cancer. Compared to the suppressive tumor immune microenvironment in advanced-stage disease, there are still immune responses and immune cell infiltration in early-stage EGFR-mutated lung cancer.^65,66^

The 13 ITT lesions that underwent surgical resection were all pathologically diagnosed as malignant (lung adenocarcinoma), indicating that our evaluation of the nodules’ nature before enrollment was credible. As a non-invasive therapy, PD-1 inhibition might be a potential treatment option for early-stage lung cancer patients and reduce the second-operation rate for patients with multifocal lesions, especially those inoperable patients with unresected high-risk GGOs. Previous studies proved that chemotherapy is ineffective on pulmonary GGO lesions.^67,68^ Therefore, to further improve the efficacy, it is worth exploring immunotherapy combined with tumor vaccine or cell therapy in GGO-featured early-stage lung cancer patients.

Our study had some limitations. This study is monocentric, with a small number of patients and a brief follow-up period; the overall survival information is absent, and a randomized clinical trial is warranted to better define the efficacy of immunotherapy in MPLC; the nature of enrolled high-risk GGOs has not been histologically diagnosed, and was evaluated by MDT according to the Lung-RADS classification and the follow-up information of lesions; the pre- and post-treatment tissue specimens were not acquired in all patients, thereby we performed relevant detection based on blood samples to verify the activity of sintilimab in responders; the driver mutations status of patients’ intent-to-treat lesions was not confirmed before enrollment; all participants received sum to 4 doses of sintilimab treatment in our study, it is worth exploring in coming work whether the extension of medication cycles can benefit more patients.

In conclusion, this study provided evidence that PD-1 inhibitor had certain activity on high-risk pulmonary GGO lesions without safety concerns. Such effects were associated with specific T-cell re-distribution, EGF/CTLA-4 cytokines compensation, and regulation of metabolism pathways. The utilization of immunotherapy in patients with early-stage lung cancer merits further investigations, and its beneficiaries need to be identified by biomarkers.

## Materials and methods

### Study oversight

This study was approved by the institutional review board of The First Affiliated Hospital of Guangzhou Medical University, and registered with ClinicalTrials.gov, NCT04026841. All participants provided written informed consent before treatment, and this trial adhered to all relevant ethical considerations. The present study was designed and the manuscript was written by the authors, who vouch for the accuracy and completeness of the data reported and adherence to the study protocol.

### Patients

Patients were eligible for enrollment if they were aged 18 years or older, and met the following criteria: (I) There were two or more nodules that cannot be resected simultaneously in the lung, and of them one or more nodules have been pathologically confirmed (surgery or fine needle aspiration) to be NSCLC; (II) At least one unresected GGO lesions (pure-GGO or GGO-predominant) with a diameter of 1–3 cm and persistent for ≥1 year, which were evaluated as high-risk (Lung-RADS category 4 or had progressed within 6 months) pulmonary nodules and suspected as primary lung cancer consistently by the multidisciplinary team (MDT, including oncologists, radiologists and pathologists); meanwhile, to assist in the diagnosis of MDT, we used an original artificial intelligence (AI) software based on radiomics to predict the nature (probability of malignancy) and EGFR mutation status (probability of positive) of the ITT lesions, with a 0.5 as the cut-off value of both two predictions.^69^ For the enrolled patients, ITT lesions were defined as the pure-GGO or GGO-predominant nodules which met the inclusion criteria, and NITT lesions referred to the pure-solid or solid-predominant nodules; both were assessed to be malignant by MDT. All patients had an Eastern Cooperative Oncology Group performance-status score of 0 or 1, normal organ function, and measurable lesions.^70^ Patients’ enrollment was consecutive and unselected by the investigators.

Patients were excluded if they had any one of the following conditions: distant metastasis, immunodeficiency, active autoimmune disease, a history of autoimmune disease, ongoing systemic immunosuppressive therapy, received treatment of other antitumor drugs within 4 weeks, had a malignancy within the previous 5 years.

### Treatments and endpoints

After baseline information collection, patients received intravenous drip sintilimab 200 mg per 3 weeks for 4 cycles; blood sampling before every cycle and chest CT scan after every 2 cycles.

The primary endpoint was the objective response rate (ORR), including complete response (CR) and partial response (PR); the calculation of ORR was based on the number of included patients. For patients with multiple ITT lesions, we take each nodule separately to evaluate, and it would be considered a responded case if one of the lesions shrunk. All patients underwent CT re-examination regularly (per 6–12 months) since completion of medication and were followed up for 3 years.

The secondary endpoint was the safety of treatment, and the follow-up for safety was continued to 3 months after the last medication. Treatment-related adverse events (TRAEs) were assessed by the National Cancer Institute Common Terminology Criteria for Adverse Events, version 5.0.^71^

Furthermore, the immune dynamics of patients during PD-1 blockade treatment were analyzed, with the examination of T/B/NK-cell subpopulations, T-cell receptor sequencing (TCR-seq), cytokines (Supplementary Table 4), exosomal RNA, and multiplexed immunohistochemistry (mIHC).

### Study design

We used a Simon’s optimal two-stage design to assess ORR as the primary endpoint. Based on previous studies, the ORR of sintilimab on GGO-featured lung cancer in our study was assumed to be at least 20%.^5,72,73^ The null hypothesis was ORR 5% versus the alternative ORR 20%. A Simon’s two-stage design, optimal version, was applied. Type I (α) and type II (β) error rates were set at 0.05 and 0.2, respectively. Accordingly, 10 cases should be enrolled in the first stage. If none of them responded, the trial would be terminated; if one or more cases responded, 19 cases would be enrolled subsequently in the second stage. Assuming a drop-out rate of 20%, a total of 36 patients needed to be included in this study.

### Radiological and pathological assessments

In this study, all included patients had received at least one CT follow-up ≥1 year prior to the enrollment, and also, a chest CT within 1 month was needed as the baseline information. The changes in size of lesions were evaluated by 2 radiologists (JCS and SX) according to the Response Evaluation Criteria in Solid Tumors (RECIST), version 1.1.^74^ The lesions would be considered as responded when the shrinkage was ≥30% after treatment.

All patients’ resected primary tumor before enrollment underwent baseline tumor staging including histodiagnosis and pathological evaluation of mediastinal lymph nodes, in terms of the criteria of the American Joint Committee on Cancer (eighth edition).^6^

### MPLC diagnosis

The occurrence of two or more primary lung cancers in the same individual is known as MPLC.^75,76^ For patients with more than one site of lung cancer, distinguishing between MPLC and intrapulmonary metastasis (IPM) is crucial in clinical practice.^77^ The judgment of MPLC is commonly based on a multidisciplinary team (MDT), taking into account clinical, radiologic, and (if available) tumor cytologic/histologic/genetic features. In this study, the included MPLC patients were diagnosed by the MDT, according to the diagnostic criteria from ACCP guidelines.^78^

### T/B/NK-cell subpopulations

The T-cell, B-cell, and NK-cell subpopulations in the peripheral blood were detected in enrolled patients using flow cytometry: 1) Take the antibody reagents from 20uL CD3/CD8/CD45/CD4 Assay Kit (Agilent) or CD3/CD16 + CD56/CD45/CD19 Assay Kit (Agilent) at room temperature, and add them to the FACS tube; 2) add 50uL of fully mixed anticoagulated peripheral whole blood to the tube; 3) gently shake the tube for 5 s using a vortex mixer and incubate for 15 min (18–25 °C); 4) add 450 uL hemolysin; 5) gently shake it for 5 s using a vortex mixer and incubate for 15 min (18–25 °C). Then the samples were analyzed using the flow cytometer (Agilent NovoCyte) and software (NovoExpress).

In this examination, CD8^+^ T cells showed CD45^+^CD3^+^CD8^+^; CD4^+^ T cells showed CD45^+^CD3^+^CD4^+^; B cells showed CD45^+^CD3^−^CD19^+^; and NK cells showed CD45^+^CD3^−^CD56^+^.

### TCR-seq, cytokines, exosomal RNA and mIHC

We performed the examination of TCR-seq, cytokines, and exosomal RNA by using blood samples of responders and non-responders. The mIHC analysis was performed on their resected tumors.

### TCR-seq

PBMCs from blood samples of patients were isolated by Ficoll density gradient centrifugation and extracted total RNA using the RNeasy Plus Mini kit (Qiagen). The total RNA (500 ng) of each sample was amplified through multiplex PCR (mPCR) using the Oncomine™ TCR Beta‑SR Assay Kit (Thermo) according to the manufacturer’s instructions. Further, TCR libraries were quantified using the Ion Library TaqMan® Quantitation Kit (Thermo) and sequenced by the Ion GeneStudio™ S5 System (Thermo). The data analysis was performed using R v.4.1.3.

### Cytokines

Serum cytokines were detected through the Cytokine/Chemokine/Growth Factor 45-Plex Human ProcartaPlex Panel 1 (Thermo) and Immuno-Oncology Checkpoint 14-Plex ProcartaPlex Panel 1 (Thermo) according to manufacturer’s instructions, which included 45 cytokines and 14 immune checkpoints, respectively. The results were measured and analyzed by the Luminex-200 system (Lumiex).

### Exosomal RNA

Blood exosomes were isolated by SEC (size exclution chromatography) methods.^79^ In brief, blood exosomes were eluted and purified using the Exosupur® columns (Echobiotech), then concentrated by 100 kDa molecular weight cut-off Amicon® Ultra spin filters (Merck). The exosomes were verified using nanoparticle tracking analysis (NTA), transmission electron microscopy (TEM) and Western blot analysis. Exosome RNA was extracted and purified with QIAGEN RNeasy Mini Kit (Qiagen). RNA concentration and purity were evaluated using RNA Nano 6000 Assay Kit of the Agilent Bioanalyzer 2100 System (Agilent Technologies). A total amount of 250pg–10ng RNA per sample was used as input material for sequencing libraries using the SMARTer Stranded Total RNA-Seq Kit (Takara Bio) and the index codes were added to attribute sequences for each sample. Library quality was assessed by the Agilent Bioanalyzer 2100 and qPCR. The libraries were then sequenced on an Illumina Hiseq platform, and paired-end reads were generated.

### mIHC

There were two panels of 10 biomarkers examined in this study, including panel 1: CD8 (cytotoxic T cells; Clone SP16; ZA0508; Zsbio), CD4 (T helper cells; Clone EP204; ZA0519; Zsbio), PD-1 (programmed cell death-1; Clone UMAB199; ZM0381; Zsbio), PD-L1 (programmed cell death-Ligand 1; Clone SP142; ZA0629; Zsbio), Foxp3 (regulatory T cells; Clone 236A/E7; ab20034; Abcam); and panel 2: CD19 (B cells; Clone UMAB103; ZM0038; Zsbio), CD56 (natural killer cells; Clone UMAB83; ZM0057; Zsbio), CD68 (macrophages; Clone KP1; ZM0060; Zsbio), CD163 (M2 macrophages; Clone 10D6; ZM0428; Zsbio), Cytokeratin (tumor cells; Clone AE1/AE3; ZM0069; Zsbio).

Formalin-fixed and paraffin-embedded (FFPE) samples were cut from surgical specimens, sections of 4 μm thickness. The slides were stained manually according to the instruction using the Opal seven-color IHC Kit (NEL797B001KT; PerkinElmer), including fluorophores 4’,6-diamidino-2-phenylindole (DAPI), Opal 650 (CD8), Opal 570 (CD4), Opal 690 (PD-1), Opal 620 (PD-L1), Opal 520 (Foxp3); Opal 620 (CD19), Opal 520 (CD56), Opal 650 (CD68), Opal 570 (CD163), Opal 690 (Cytokeratin), and TSA Coimarin system (PerkinElmer). Every staining round contained a slide of tonsil as positive control. Stained slides were scanned by the Vectra (Vectra 3.0.5; PerkinElmer). After scanning, a selection of 15 representative images were used to analysis by the inform software (inform 2.3.0; PerkinElmer).

### Statistical analysis

The Student’s *t* test, Wilcoxon’s rank-sum test, and ANOVA were applied to compare continuous variables, including the proportion and absolute counting of circulating immune cells (T/B/NK-cell), the ratio of CD8^+^/CD4^+^ T-cell, the TCR clonality and diversity (Shannon-index/evenness/convergence), and the concentration values of various cytokines between responders and non-responders (T1–T4). The ORR and TRAEs were expressed as frequencies and percentages.

To compare the changing trend of proportion of various immune cells over treatment between responded and non-responded patients, the repeated measures analysis of variance was applied, with the change of various indicators as the dependent variable, and response, time, and the response×time interaction as the independent variables.

A two-sided *p*-value < 0.05 was considered as significant. Clinical analyses were conducted using SPSS Statistics (version 23.0, IBM), R v.4.1.3, Microsoft Excel v.2019 and GraphPad Prism v.8.00. The specific parameters for R v.4.1.3 analyses used in this study were described in the Supplementary materials.

### Supplementary information

Supplementary Materials

Study protocol

## Data Availability

The data supporting the findings of this study are available on request from the corresponding author Wenhua Liang, Email: liangwh1987@163.com. All the sequencing data have been deposited at the Gene Expression Omnibus (GEO, https://www.ncbi.nlm.nih.gov/geo/), which is hosted by the NCBI under the accession code GSE260770.
