# Supplementary material for: The activity and immune dynamics of PD-1 inhibition on high-risk pulmonary ground glass opacity lesions: insights from a single-arm, phase II trial
Source: Signal Transduct Target Ther. 2024 Apr 19;9:93. doi: 10.1038/s41392-024-01799-z (PMC11026465; doi:10.1038/s41392-024-01799-z)
Supplement: Supplementary file 1 — Supplementary Materials [file 41392_2024_1799_MOESM1_ESM.docx]

Supplementary Materials for

The activity and immune dynamics of PD-1 inhibition on high-risk pulmonary ground glass opacity lesions: insights from a single-arm, phase II trial

Bo Cheng^1^*, Caichen Li^1^*, Jianfu Li^1^*, Longlong Gong^2^*, Peng Liang^1^*, Ying Chen^1^, Shuting Zhan^1^, Shan Xiong^1^, Ran Zhong^1^, Hengrui Liang^1^, Yi Feng^1^, Runchen Wang^1^, Haixuan Wang^1^, Hongbo Zheng^2^, Jun Liu^1^, Chengzhi Zhou^1^, Wenlong Shao^1^, Yuan Qiu^1^, Jiancong Sun^3^, Zhanhong Xie^4^, Zhu Liang^5^, Chenglin Yang^6^, Xiuyu Cai^7^, Chunxia Su^8^, Wei Wang^1^, Jianxing He^1^†, Wenhua Liang^1^†.

*Joint first authors; †Joint corresponding authors

Correspondence to:

Wenhua Liang, MD, Email: [liangwh1987@163.com](mailto:liangwh1987@163.com); Jianxing He, MD, Email: [drjianxing.he@gmail.com](mailto:drjianxing.he@gmail.com).

**This PDF file includes:**

Figures S1 to S8

Tables S1 to S4

The parameters for the analyses using R v.4.1.3

**Other Supplementary Materials for this manuscript include the following:**

Study protocol

**Supplementary Figure 1. The nature (probability of malignancy) and EGFR mutation status (probability of positive) of the 49 ITT lesions predicted by AI software based on baseline CT of 36 enrolled patients.**

^*^ Patients 5, 18, and 34 were unable to make predictions due to baseline CT not meeting the required DICOM format; additionally, the EGFR mutation status prediction could only be performed when the malignant probability of nodules was not less than 0.3.


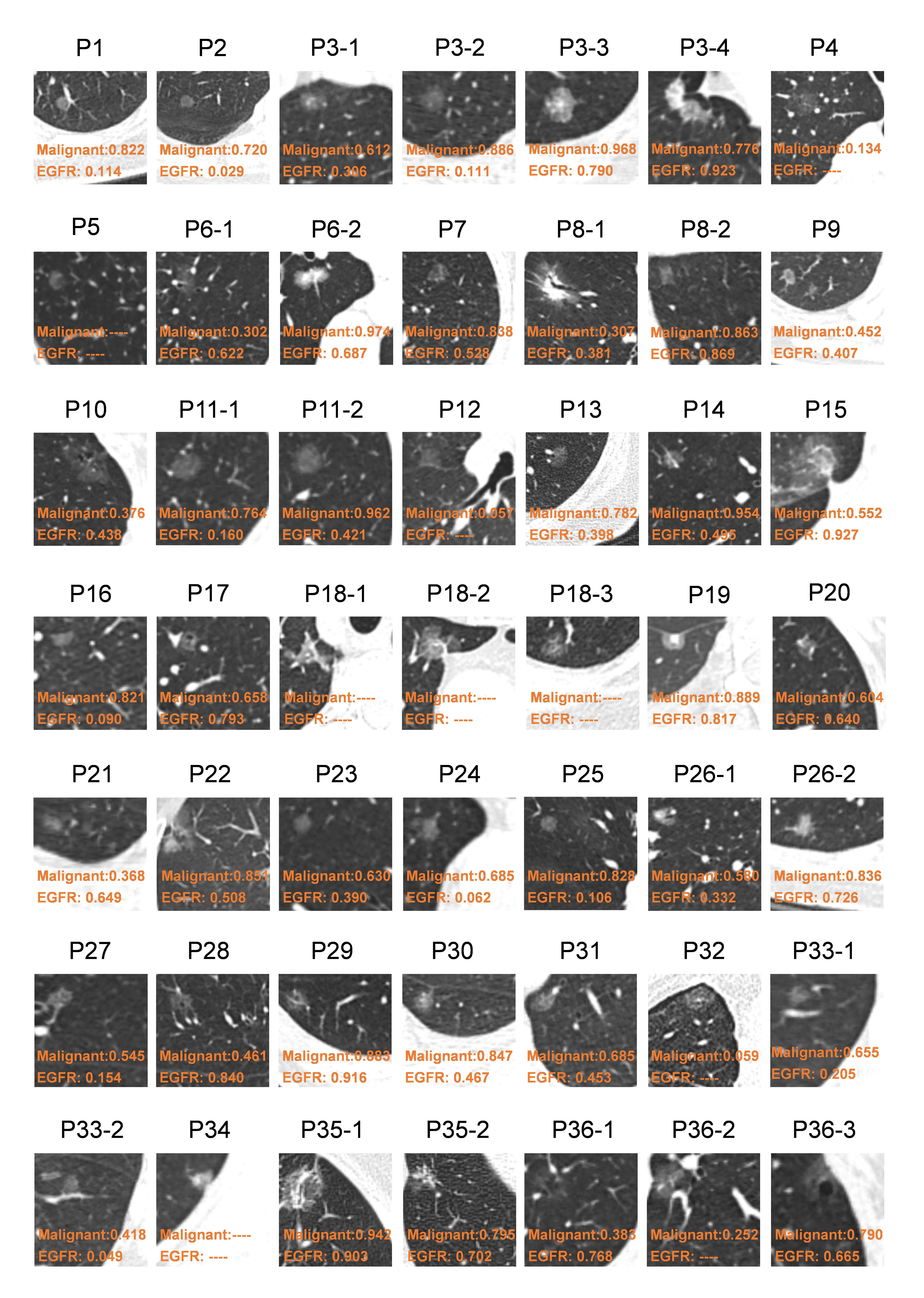


**Supplementary Figure 2. The mIHC analysis based on 1 responded and 5 non-responded tumor issues.**

(a-b) These structures could be visible by staining, including panel I (a): CD8^+^ T cells (green), CD4^+^ T cells (yellow), PD-1^+^ cells (magenta), PD-L1^+^ cells (orange), Foxp3^+^ regulatory T cells (red); and panel II (b): CD19^+^ B cells (green), CD56^+^ NK cells (red), CD68^+^ macrophages (yellow), CD163^+^ M2 macrophages (cyan), cytokeratin^+^ tumor cells (magenta).


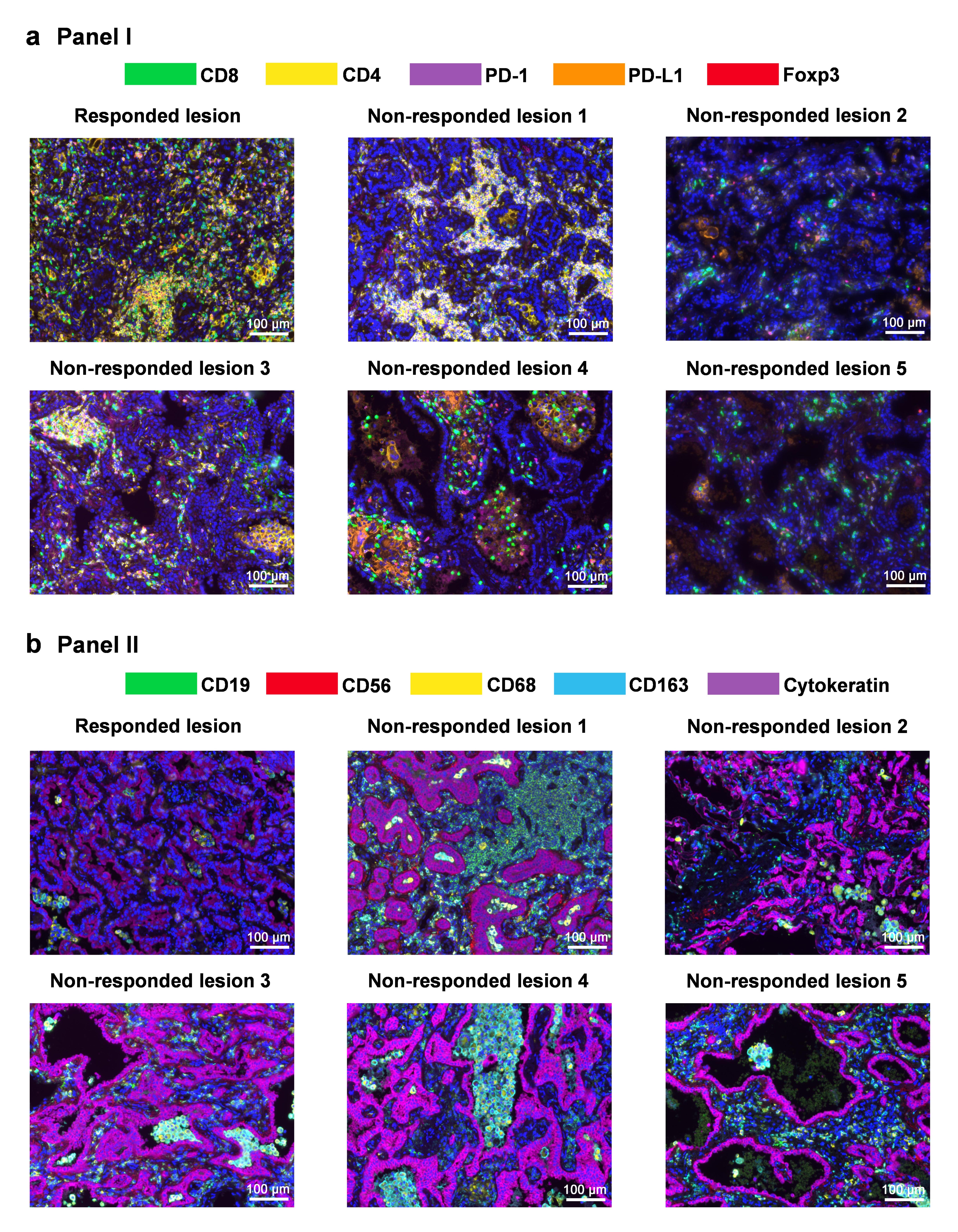


**Supplementary Figure 3. Clinical course and timing of surgery of the patients receiving mIHC detection.**

The pathology results (Invasive adenocarcinoma, IA; Minimally invasive adenocarcinoma, MIA) of the resected tumor, the time interval from the primary surgery (P) to starting sintilimab therapy, and the time interval from sintilimab treatment completion to the second surgery (S) were provided. All these patients underwent 4 doses of sintilimab treatment (200 mg per 3 weeks).


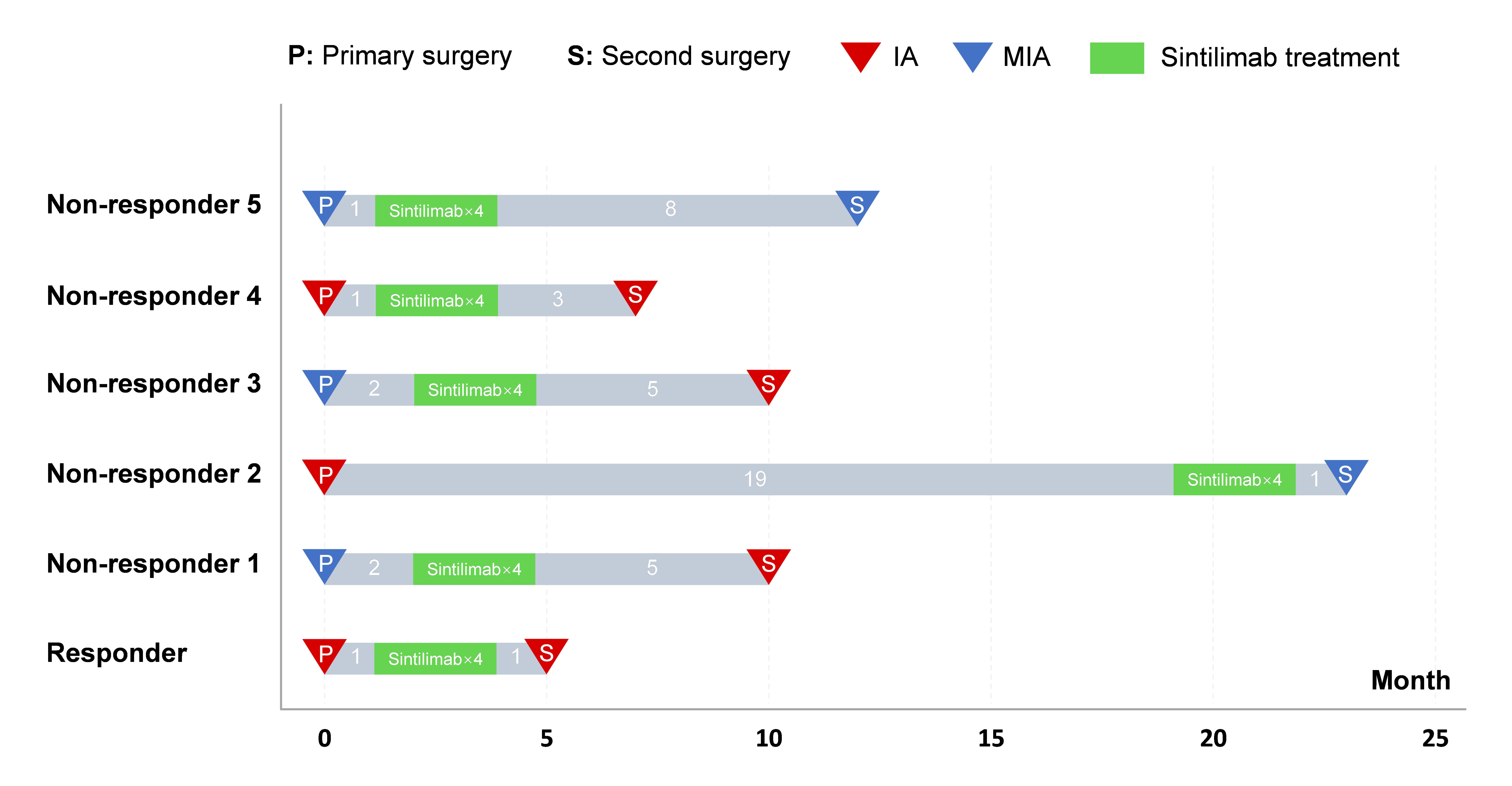


**Supplementary Figure 4. T cell detection of responders and non-responders.**

(a-b) The absolute counting of CD8^+^(a) and CD4^+^ T cell (b) in the baseline blood (T1) of responders (red, n=5) and non-responders (blue, n=31).

(c-d) From T1 to T4, the changing trend of absolute counting of CD8^+^ (c) and CD4^+^ T cell (d) in responders (red, n=5) and non-responders (blue, n=31).


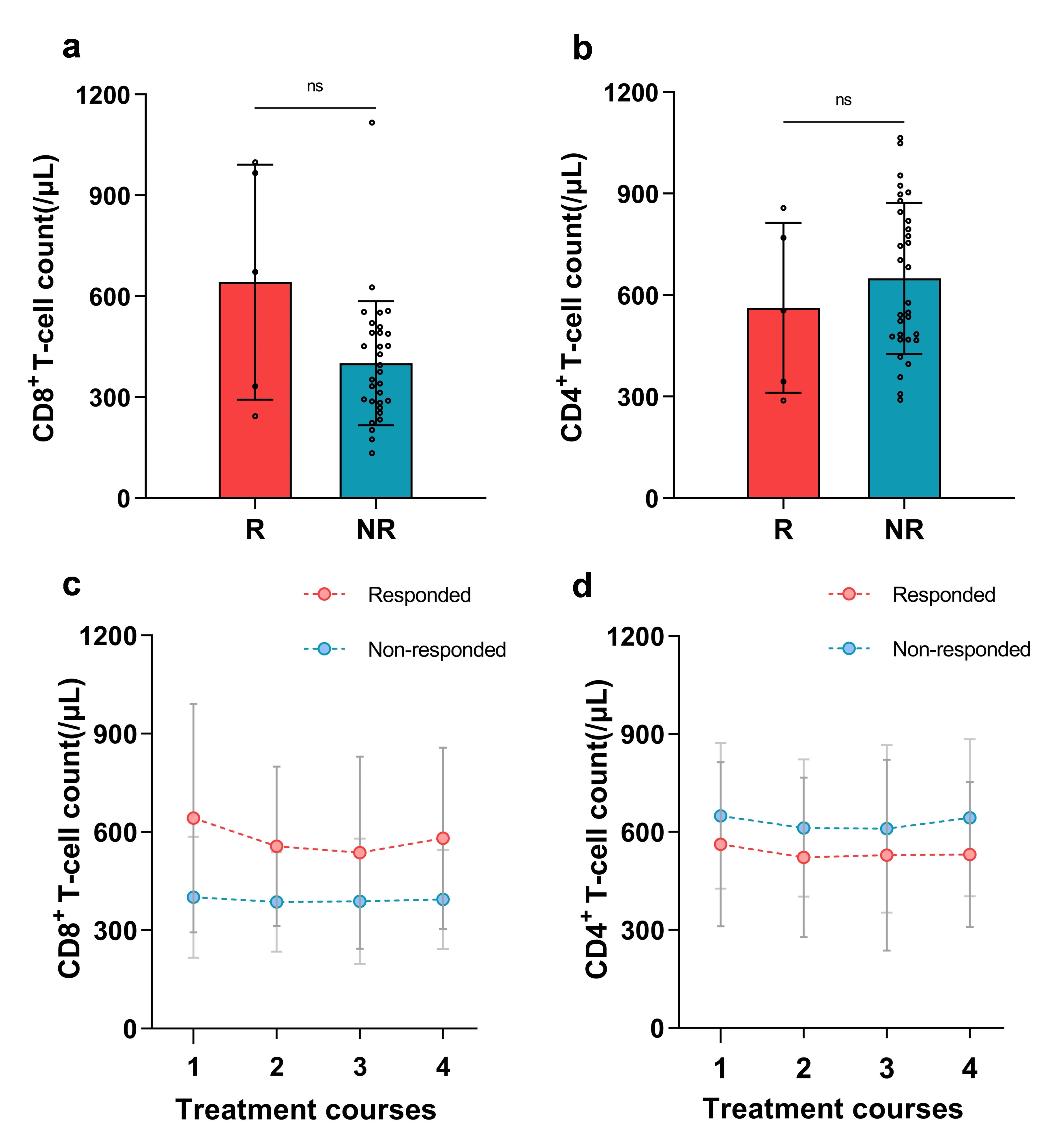


**Supplementary Figure 5. The changes of various immune cells of each enrolled patient (N=36) over time.**

(a-d) The changing trend of proportion of CD8^+^ T cell (a), CD8^+^/CD4^+^T-cell (b), B-cell (c), NK-cell (d) over time (T1-T4) in each responder (red line, n=5) and non-responder (blue line, n=31);

(e-f) The changing trend of CD8^+^ T cell count (e) and CD4^+^ T cell count (f) over time (T1-T4) in each responder (red line, n=5) and non-responder (blue line, n=31).


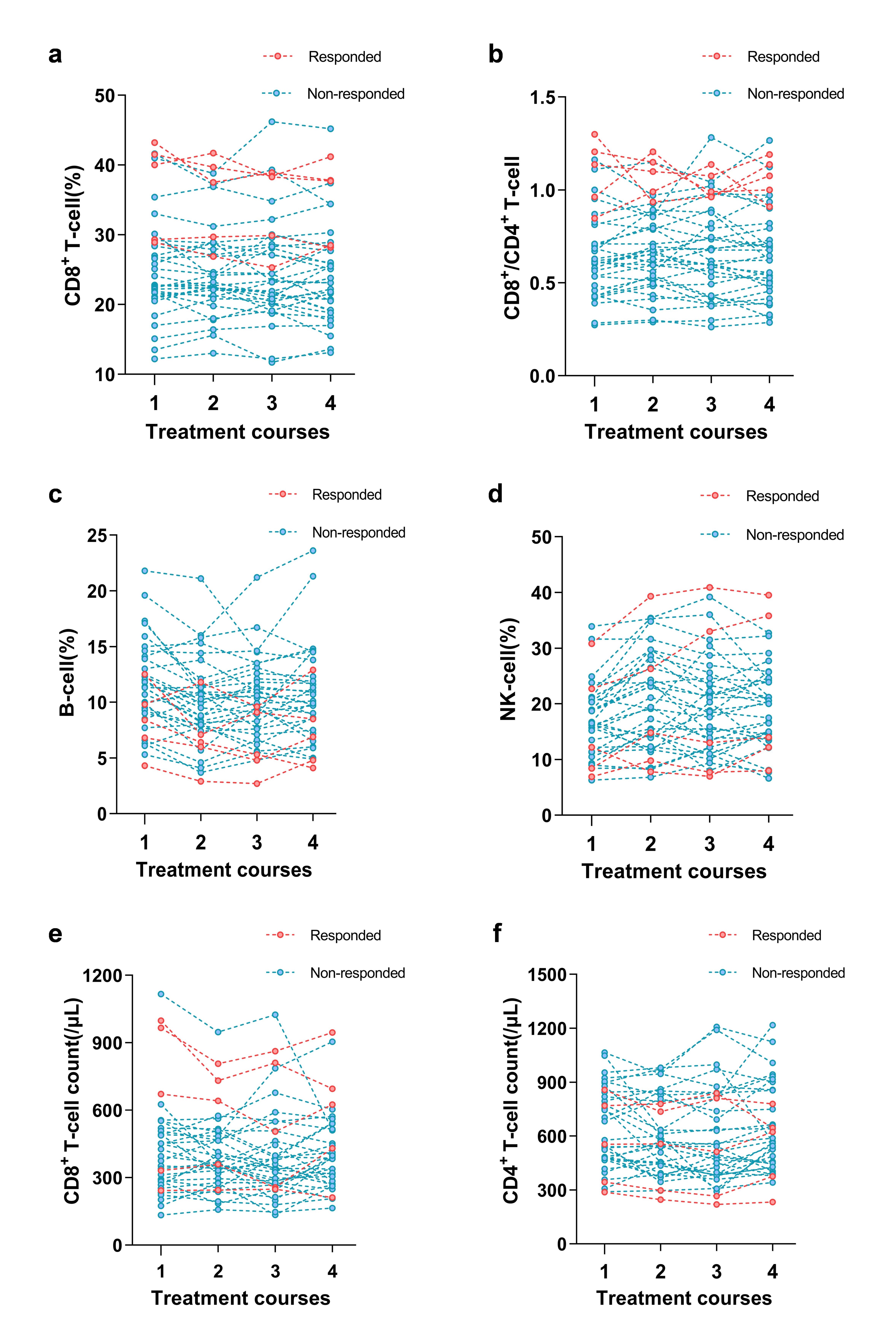


**Supplementary Figure 6. The concentration values of various cytokines of patients at different time point.**

The concentration values of various cytokines between group-R (orange, n=5) and group-NR (blue-grey, n=5) patients were compared at T1 (a), T2 (b), T3 (c), and T4 (d) time point, respectively.





**Supplementary Figure 7. The results of time-series clustering analysis in group-R patients (n=5).**


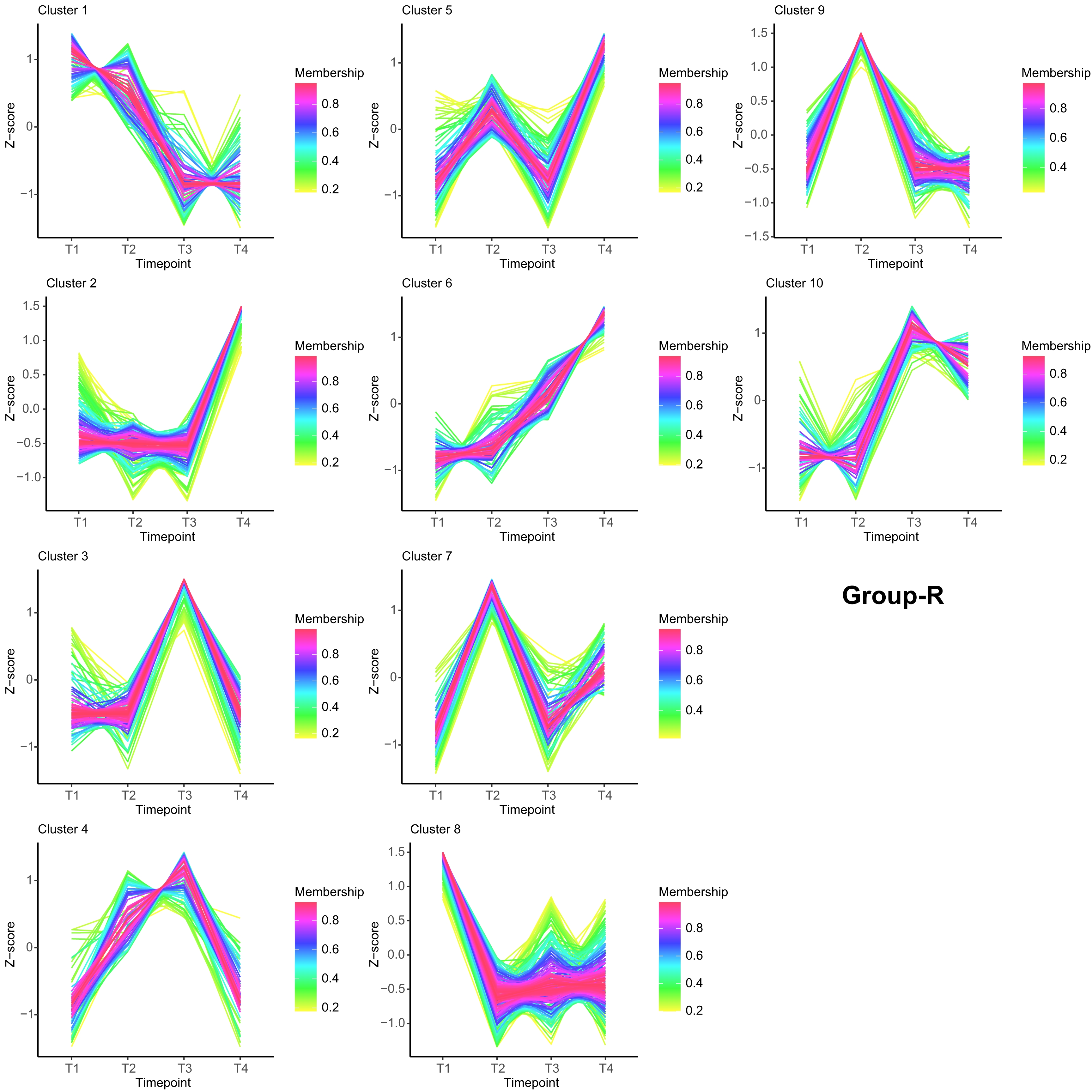


**Supplementary Figure 8. The results of time-series clustering analysis in group-NR patients (n=5).**


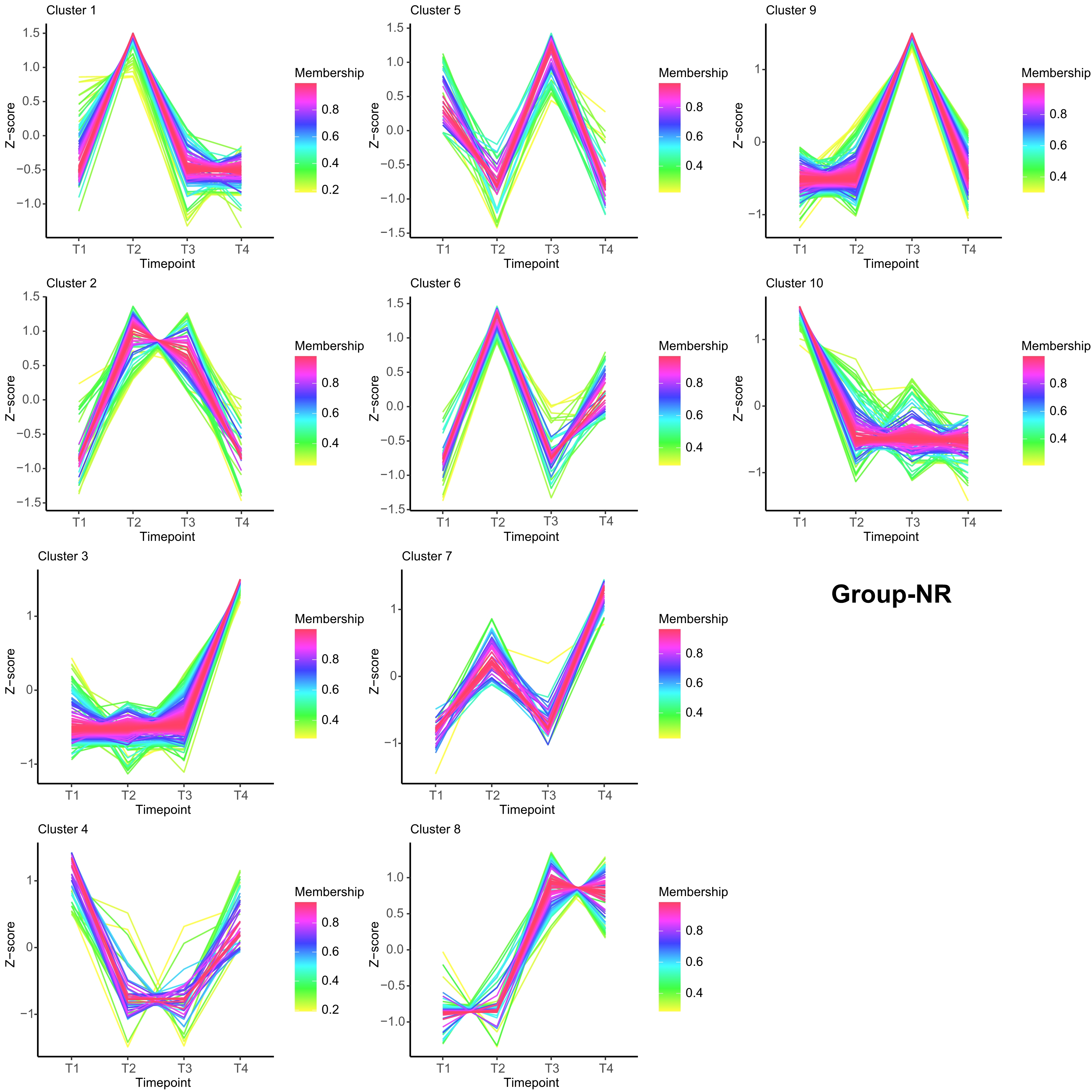


**Supplementary Table** **1. Surgical information of the second surgery after sintimab treatment in enrolled patients.**

| Patient-lesion | Lesion type | Radiological  type | Diagnosis | Gene mutation | Surgery | Lymph node dissection | Time interval  (months)^*^ |
| --- | --- | --- | --- | --- | --- | --- | --- |
| 1 | ITT lesion | Mixed GGO | MIA | EGFR 19 del | Wedge resection | No | 1 |
| 2 | ITT lesion | Mixed GGO | MIA | EGFR 19 del | Wedge resection | No | 1 |
| 3-1 | ITT lesion | Pure GGO | MIA | EGFR 21 L858R | Segmentectomy | Yes | 1 |
| 3-2 | NITT lesion | Solid-dominant | IA | EGFR 19 del |  |  |  |
| 4 | ITT lesion | Pure GGO | MIA | Not available | Wedge resection | No | 5 |
| 5 | ITT lesion | Mixed GGO | MIA | EGFR 20 insert | Wedge resection | No | 1 |
| 6 | ITT lesion | Mixed GGO | MIA | Not available | Lobectomy | No | 4 |
| 7-1 | ITT lesion | Mixed GGO | MIA | Not available | Segmentectomy | Yes | 5 |
| 7-2 | ITT lesion | Mixed GGO | IA | EGFR 19 del |  |  |  |
| 8 | ITT lesion | Mixed GGO | MIA | Not available | Wedge resection | No | 15 |
| 9 | ITT lesion | Mixed GGO | IA | EGFR 20 insert | Segmentectomy | Yes | 6 |
| 10 | ITT lesion | Mixed GGO | MIA | EGFR 19 del | Wedge resection | Yes | 8 |
| 11 | ITT lesion | Mixed GGO | MIA | EGFR 20 insert | Wedge resection | No | 6 |
| 12 | ITT lesion | Mixed GGO | IA | EGFR 21 L858R | Segmentectomy | Yes | 3 |

^*^The time interval between the sintilimab treatment completion and the second surgery.

**Supplementary Table 2. The proportions (%) of various immune cells observed in the mIHC.**

| Tumors | CD8^+^ | CD4^+^ | CD19^+^ | CD163^+^ |
| --- | --- | --- | --- | --- |
| Responded 1 | 19.554 | 8.276 | 0.571 | 5.884 |
| Non-responded 1 | 11.593 | 19.049 | 5.852 | 9.816 |
| Non-responded 2 | 2.208 | 3.534 | 0.158 | 1.691 |
| Non-responded 3 | 10.092 | 19.179 | 2.442 | 7.243 |
| Non-responded 4 | 10.751 | 3.782 | 0.933 | 4.697 |
| Non-responded 5 | 10.774 | 5.867 | 0.664 | 7.277 |
| Mean-NR (1-5) ^*^ | 9.08±3.47 | 10.28±7.26 | 2.00±2.07 | 6.14±2.75 |

**^*^**NR, non-responded.

**Supplementary Table 3. The baseline information of 10 patients** **(5 responded and 5 non-responded) who received the TCR-seq, cytokines, and exosomal RNA examinations.**

| **Responder** | **Sex** | **Age**  **(years)** | **Smoking history** | **Non-responder** | **Sex** | **Age**  **(years)** | **Smoking history** |
| --- | --- | --- | --- | --- | --- | --- | --- |
| Patient 1 | Male | 65 | Yes | Patient 1 | Male | 59 | Yes |
| Patient 2 | Female | 69 | No | Patient 2 | Female | 66 | No |
| Patient 3 | Female | 69 | No | Patient 3 | Female | 71 | No |
| Patient 4 | Male | 78 | Yes | Patient 4 | Male | 57 | Yes |
| Patient 5 | Male | 58 | No | Patient 5 | Female | 74 | No |

**Supplementary Table 4.** **List of 45 cytokines and 14 immune checkpoints examined in this study.**

| Cytokines | | | | | |
| --- | --- | --- | --- | --- | --- |
| 45-plex | | | | | |
| GM-CSF | IL-2 | IL-6 | IL-18 | IFN-gamma | IL-4 |
| IL-12p70 | TNF-alpha | IL-1beta | IL-5 | IL-13 | IL-9 |
| IL-17A | IL-22 | IL-27 | IL-10 | IL-21 | IL-23 |
| IFN-alpha | IL-1RA | IL-15 | TNF-beta | IL-1alpha | IL-7 |
| IL-31 | Eotaxin | GRO-alpha | IL-8 | IP-10 | MCP-1 |
| MIP-1alpha | MIP-1beta | SDF-1alpha | RANTES | BDNF | HGF |
| SCF | NGF-beta | LIF | VEGF-A | VEGF-D | EGF |
| PDGF-BB | FGF-2 | PlGF |  |  |  |
| 14-plex | | | | | |
| BTLA | GITR | HVEM | IDO | LAG-3 | PD-1 |
| PD-L1 | PD-L2 | TIM-3 | CD28 | CD80 | CD137 |
| CD27 | CD152 |  |  |  |  |

**Parameters for the analyses using R v.4.1.3**

***Clustering analysis for TCR***

The heatmap of clustering analysis for TCR was performed using “pheatmap” package, with the following parameters: clustering_method= “mcquitty”, cluster_rows= TRUE, cluster_cols= TRUE.

***Time-series clustering analysis for gene expression***

The time-series clustering analysis for gene expression was performed using “TCseq” package, with the following parameters: algo (clustering method) = “cm(cmeans)”, k= “10”, dist= “euclidean”, standardize (z-score) = “TRUE”.
