# Supplementary material for: The activity and immune dynamics of PD-1 inhibition on high-risk pulmonary ground glass opacity lesions: insights from a single-arm, phase II trial
Source: Signal Transduct Target Ther. 2024 Apr 19;9:93. doi: 10.1038/s41392-024-01799-z (PMC11026465; doi:10.1038/s41392-024-01799-z)
Supplement: Supplementary file 2 — Study protocol [file 41392_2024_1799_MOESM2_ESM.pdf]

The First Affiliated Hospital of Guangzhou Medical University

Protocol

Center: The First Affiliated Hospital of Guangzhou Medical University, National Clinical Research Center for Respiratory Disease, Guangzhou Institute of Respiratory Health, State Key Laboratory of Respiratory Disease.

Version Date: 13-July-2019

Title: The Efficacy of PD-1 Antibody Sintilimab on Ground Glass Opacity Lesions in Patients with Early-stage Multiple Primary Lung Cancer, a single-arm, phase II study (CCTC-1901)

Principal Investigator:

Wenhua Liang, MD, Email: liangwh1987@163.com;

Jianxing He, MD, Email: drjianxing.he@gmail.com.

Department of Thoracic Surgery and Oncology, the First Affiliated Hospital of Guangzhou Medical University.

151, Yanjiang Road, Guangzhou 510120, China.

Tel: +86-20-83337792; Fax: +86-20-83350363.

Statistician: Peng Liang

Department of Thoracic Surgery and Oncology, the First Affiliated Hospital of Guangzhou Medical University.

151, Yanjiang Road, Guangzhou 510120, China.

Tel: +86-20-83337792; Fax: +86-20-83350363.

jpdiary@163.com

Protocol Signature Page

I confirm that I have read this protocol, I understand it, and I will work according to this protocol and to the ethical principles stated in the Declaration of Helsinki, the applicable ICH guidelines for good clinical practices, and the regulations relating to the conduct of the protocol. I have read and understand the information in the Investigators' Brochure (or Manufacturer's Brochure) regarding the risks and potential benefits. I will promptly submit the protocol to the applicable IRB for review and approval. Once the protocol has been approved by the IRB, I understand that any modification made during the course of the study must first be approved by the IRB. I certify that I, and the study staff, have received the requisite training to conduct this research protocol. I agree to maintain adequate and accurate records in accordance with the First Affiliated Hospital of Guangzhou Medical University. I agree to maintain the confidentiality of all information received or developed in connection with this protocol.

Instructions to Principal Investigator: Sign and Date this signature page and print your name. Return the original, completed and signed to the Clinical Protocol & Data Management Office. Retain a copy in the regulatory binder.

______________________________ ______________

Signature of Principal Investigator Date

____

Principal Investigator Name (Print)

____

Name of Institution

Protocol Synopsis

**Title**	The Efficacy of PD-1 Antibody Sintilimab on Ground Glass Opacity (GGO) Lesions in Patients with Early-stage Multiple Primary Lung Cancer, a single-arm, phase II study
**Study ID**	CCTC-1901
**Version**	1.1
**Principal Investigator**	Jianxing He
**Center**	The First Affiliated Hospital of Guangzhou Medical University
**Design**	A single center, single-arm, phase II study using the Simon's optimal two-stage design
**Object of study**	Early-stage multiple primary lung cancer (MPLC) presenting as GGO
**Study Agent**	Sintilimab 200mg, IV, Q3W
**Inclusion criteria**	1) Above 18 years of age;<br>2) Two or more GGO lesions (pure GGO or GGO-predominant) that cannot be resected simultaneously;<br>3) There was at least one lesion with a diameter of 1-3cm pathologically confirmed as lung cancer;<br>4) ECOG PS 0-1 score;<br>5) The function of vital organs meets the following requirements:<br>Absolute counting of neutrophil ≥1.5×109/L;<br>Platelet ≥90×109/L;<br>Hemoglobin ≥9g/dL;

	Thyroid-stimulating hormone (TSH) ≤ULN;<br>Bilirubin ≤ULN;<br>ALT and AST≤1.5 ULN;<br>AKP≤2.5 ULN;<br>Serum creatinine ≤ 1.5 ULN or creatinine clearance⩾60mL/min<br>6) Women of childbearing age must already be using reliable contraception or have had a pregnancy test (serum or urine) with a negative result within 7 days prior to inclusion and be willing to receive appropriate method of contraception during the trial and 8 weeks after the last trial drug administration. For males, using appropriate methods of contraception or surgical sterilization are needed during the trial period and 8 weeks after the last course of medication;<br>7) The participants voluntarily joined the study and signed the informed<br>consents. The participants had good compliance and cooperated with follow-up visits.
**Exclusion criteria**	Patients will be excluded if they meet one of below conditions:<br>1) Non-calcified lesions with a diameter of > 3cm were present<br>2) Patients with distant metastases were excluded<br>3) The presence of any active autoimmune disease or a history of autoimmune disease (as follows, but not limited to: autoimmune hepatitis, interstitial pneumonia, uveitis, enteritis, hepatitis, hypophysitis, vasculitis, nephritis, hyperthyroidism, reduced thyroid function; Participants who had vitiligo or had complete remission of asthma in childhood could be included without any intervention as adults; Asthma in which participants require medical intervention with bronchodilators is not included);<br>4) Patients who have used other anti-tumor drugs in clinical study

	within 4 weeks;<br>5) Patients with severe allergic reaction to monoclonal antibody;<br>6) Clinical symptoms or diseases of the heart that are not well controlled, such as: a. NYHA grade 2 or above heart failure; b. Unstable angina pectoris; c. Myocardial infarction within 1 year; d. Clinically significant supraventricular or ventricular arrhythmias requiring treatment or intervention;<br>7) Participants with congenital or acquired immune deficiency (such as HIV infected persons), or active hepatitis (hepatitis B reference: HBsAg positive, HBV DNA≥2000 IU/ml or copy number ≥104/ml; Hepatitis C reference: HCV antibody positive;
**Exit criteria**	1) The participant withdraws informed consent and requires withdrawal;<br>2) During the enrollment period, participants were required to undergo surgical resection of target lesions;<br>3) Imaging examination showed the progression of the disease;<br>4) Those who cannot tolerate toxicity;<br>5) The participants lost follow-up or had positive HCG;<br>6) Other situations in which the investigator deems it necessary to withdraw from the study.
**Termination criteria**	1) Finding that there is an unexpected, significant or unacceptable risk to the participant;<br>2) The investigational drug/trial treatment does not work, or it is pointless to continue the trial;<br>3) The applicant decides to terminate the study due to reasons such as significant delay in participant inclusion or frequent protocol deviations.

**Study endpoint**	Primary endpoint:<br>Objective response rate (ORR)<br>Secondary endpoint:<br>The safety of sintilimab
**Sample size**	According to previous studies, the ORR of anti-PD-1 antibody monotherapy for non-small cell lung cancer (NSCLC) was assumed to be 20%, and the ORR of routine observation was not more than 5%. According to the optimal design principle of Simon Phase II trial, α=0.05 (bilateral), β=0.2, the sample size of phase I was calculated to be 10 cases. If no one case was effective, the trial was terminated. If 1 case or more is effective, then enter the second stage, continue to enroll 19 cases, combined with the first stage, a total of 29 cases, according to the 20% of dropping out rate, the study needs to enroll 36 cases.
**Therapeutic regimen**	Sintilimab of 200mg was administered intravenously per 3 weeks, and the efficacy was evaluated after the second and fourth treatment cycles.
**Statistical analysis**	The Student's t-test, Wilcoxon's rank-sum test, and ANOVA were applied to compare continuous variables and the χ2 test or Fisher's exact test was applied to assess the association between categorical variables. The ORR and TRAEs were expressed as frequencies and percentages. Exact tests were performed where applicable.<br>A two-sided p-value <0.05 was treated as significant.

**Timeline**	2019.09~2020.09 Enroll patients, analyze data and record data;<br>2020.10~2022.04 Follow-up and data collection of enrolled patients;<br>2022.05~2023.05 Further analyses and exploration.

Study flow charts

Phases	Screening period		Treatment period		End of treatment [21]	Safety follow-up [22] (90 days)	Follow-up of survival [23] (Per 8 weeks)
	-28-1 day	-7-1 day	Cycle 1 D1±3	Subsequent cycles D1±3			
**Baseline**							
Written informed consent [1]	X						
Inclusion/exclusion criteria	X						
Demography/past medical history/past medication [2]	X						
Vital signs [3]	X			X	X	X	
Weight/height [4]	X			X	X	X	
Physical examination [5]	X			X	X	X	
ECOG score[6]	X			X	X	X	
**Laboratory examination**							
12-leads electrocardiogram [7]	X		X	X	X	X	
Blood routine/blood biochemistry/urine routine/stool test [8]		X		X	X	X	
Coagulation function [9]		X		X	X	X	
Pregnancy tests [10]		X			X		
Thyroid function [11]	X			Per 2 cycles	X	X	
Myocardial enzyme [12]		X		Check if there are clinical signs	X		
Pituitary-adrenal axis examination [13]	X			Per 2 cycles	X		
HIV,HBV and HCV[14]	X						
Ultrasonic cardiogram [15]		X		Check if there are clinical signs	X		
Pulmonary function test [16]	X			Check if there are clinical signs			
AE evaluation [17]	X			X	X	X	

**Follow up after treatment**							
Survival state						X	X
Subsequent antitumor treatment						X	X
**Efficacy evaluation**							
Imaging evaluation[18]	X		Per 2 cycles		X		
**Biomarkers**							
Tissue specimens [19]					X		
Peripheral Blood [20]	X		Same with imaging evaluation		X		

Notes:

1. ICF signing should be performed prior to any protocol-specified procedures, except for tumor imaging and tumor tissue biopsy within the specified time limit prior to the first dose.

2. Previous medications include initial diagnostic therapy, including chemotherapy, radiotherapy and surgery, etc., and the time of the last anti-tumor therapy before that must be recorded.

3. Vital signs include temperature, pulse rate, respiratory rate, and blood pressure. It was performed during the screening period, before each sintilimab administration, at the end of treatment, and at the safety follow-up. Blood pressure monitoring: During each blood pressure measurement, smoking and coffee drinking should be prohibited within 30 minutes before the measurement, and at least 10 minutes of quiet rest should be taken. During the measurement, the elbow should be placed in the sitting position at the same level as the heart, and each blood pressure measurement should be taken on the same side. Blood pressure was measured by the investigator during the screening period and before each planned sintilimab infusion. During the study, blood pressure monitoring was completed by the participants themselves and recorded in the participants' diary card. Blood pressure was measured at least 3 times a week in the first 2 cycles, and if blood pressure was abnormal, it was tracked every day. If the blood pressure was normal, the blood pressure was checked twice a week after the third cycle.

4. Height measurements were performed only during the screening period. Weight measurements were required during the screening period, before each dose, at the end of treatment, and at safety follow-up visits.

5. Physical examinations were performed during the screening period, before each sintilimab administration, at the end of treatment, and at safety follow-up visits.

6. ECOG PS was performed during the screening period, before each sintilimab administration, at the end of treatment, and at the safety follow-up.

7. 12-lead ECG was performed during the screening period, within 30 minutes of completion of each sintilimab infusion, at the end of treatment, and at safety follow-up visits.

8. Blood routine includes: red blood cell (RBC), hemoglobin (HGB), hematocrit value (HCT), white blood cell (white blood cell), WBC), platelet (PLT), leukocyte classification (lymphocyte count (LYM), absolute neutrophil count, ANC), monocyte count (MONO), eosinophil count (EOS), basophil cell count (BASO)]. Blood biochemistry includes: Liver function [serum total bilirubin (TBIL), alanine aminotransferase (ALT), aspartate transferase (aspartate transferase, AST, γ-glutamyl transferase (γ-GT), alkaline phosphatase (ALP), albumin (ALB), total protein (TP), TP), lactic dehydrogenase (LDH)]; Renal function [blood urea nitrogen (BUN), creatinine (Cr)]; Blood electrolytes (Na, K, Cl, Mg, Ca, P); Lipase, amylase and fasting blood glucose (FBG). Routine urine tests include: PH, urinary albumin (UALB), urine protein (UPRO), urine red blood cell (URBC), urine glucose (UGLU). Screening urine routine showed negative urine protein was eligible for inclusion. Stool examination: occult blood, such as fecal occult blood + must be reexamined, reexamined fecal occult blood +, require gastrointestinal endoscopy. Routine blood tests, blood biochemistry, urine tests, and stool tests were performed during the screening period (within 7 days before the first dose of the study drug), before each dose of sintilimab, at the end of treatment, and at the safety follow-up. Inspections will be conducted at the various research centers.

9. Coagulation tests include: thrombin time (TT), prothrombin time (PT), activated partial thromboplastin time, APTT) and international normalized ratio (INR). It was performed within 7 days before the first dose of study treatment, before the administration of sintilimab b on day 1 of each cycle, at the end of treatment, and at the safety follow-up. Inspections will be conducted at the various research centers.

10. Women of childbearing age will have a serum pregnancy test within 3 days before the first dose and at the end of treatment. Inspections will be conducted at the various research centers.

11. Thyroid function tests include: triiodothyronine (T3), thyroxine (T4), free triiodothyronine (FT3), free thyroxine (free thyroxine 4, T4), FT4) and thyroid stimulating hormone (TSH). It was performed during the screening period, each cycle 2-4, at the end of treatment, and at the safety follow-up. Inspections will be conducted at the various research centers.

12. Myocardial enzyme spectrum examination: LDH, AST, Creatine kinase (CK), creatine kinase isoenzyme (CK-MB) and ALT. The examination was performed once within 7 days before the first dose of study treatment, and thereafter only when symptoms such as precardiac pain, palpitations, and ECG abnormalities were present, and at the end of treatment.

13. Pituitary adrenal axis (HPA) examination: including hypothalamic adrenocorticotropic hormone releasing hormone (CRH), pituitary adrenocorticotropic hormone (plasma ACTH) and adrenocortical hormone measurement. Adrenocortical hormone measurements included serum cortisol, urinary free cortisol (UFC), urinary 17-ketosteroid (17-KS), and urinary 17-ketosteroid (17-KGS). In eligible hospitals, monitoring is recommended during the screening period, before administration of sintilimab on day 1 of each cycle in cycles 2-4, and at the end of treatment.

14. Including HBV, HCV and HIV antibody tests, HBV test requirements: During the screening period, HbsAg was tested to determine whether HBV infection was detected, and if positive, HbsAg (quantitative), HbsAb (qualitative), HbcAb (qualitative), HbeAg (qualitative), HbeAb (qualitative) and HBV-DNA (qualitative, if positive, quantitative testing must be performed). HCV testing requirements: During screening, test for HCV-Ab to determine HCV infection, and test for HCV-RNA if positive (qualitative, or quantitative). Hepatitis B carriers participating in the study should ask the researcher to arrange antiviral treatment at their discretion.

15. Echocardiography: One examination should be performed within 7 days before the first dose and at the end of the study. This examination should be supplemented if

clinically significant ECG abnormalities occur during the study.

16. Pulmonary function test: Tidal volume (VT), respiratory rate (BE), resting ventilation per minute (MV), inspiratory volume (ERV), inspiratory volume (IC), maximal vital capacity (VCmax), forced vital capacity (FVC), forced expiratory volume in the first second (FEV1), forced expiratory volume in the first second to vital capacity ratio (FEV1/FVC), Peak flow velocity (PEF), maximal expiratory flow at 75% vital capacity (MEF75), maximal expiratory flow at 50% vital capacity (MEF50), maximal expiratory flow at 25% vital capacity (MEF25), maximal expiratory flow at 25% vital capacity (MVV), Total lung capacity (TLC), residual to total ratio (RV/TLC), ventilatory reserve function (BR), pulmonary carbon monoxide dispersion capacity in one breath (DLCO SB), airway resistance (Raw eff) and specific airway conductance (sGaw eff) were performed during the screening period and subsequent according to investigator judgment.

17. Safety assessment of AE and laboratory tests will be evaluated according to NCI CTCAE v4.03. AEs from the first dose of study drug to the end of safety follow-up and all SAEs from the signing of ICF to the end of safety follow-up should be recorded in the CRF. AE and SAE are defined, recorded, judged for relevance, judged for severity, reported time limit and treated as described in Part 9 of the protocol. AEs associated with study drugs need to be followed until remission to grade 0-1, symptom stabilization, participant withdrawal from ICF, or initiation of new antitumor therapy, whichever occurs first.

18. The imaging methods used for tumor evaluation at baseline must be consistent with those used at each subsequent follow-up evaluation, and computed tomography (CT) or magnetic resonance imaging (MRI) scans are recommended. Other examinations are performed depend on the symptoms and signs of each participant. At baseline, chest, abdominal and pelvic scans (scanning from the apex of the lung to the suprapubic symphysis), brain and bone scans, and all known or suspected disease sites must be performed. Each subsequent clinical tumor imaging evaluation should include the chest, abdomen, and pelvis. Brain and/or bone scans may be performed when clinically indicated. Researchers can increase the frequency of imaging monitoring according to

the actual situation. Baseline tumors information will be collected within 28 days before the first dose, and imaging data obtained before signing informed consent can be used for screening tumor assessment as long as protocol requirements are met. Evaluation was performed every two cycles until the end of the study or progressive disease (PD) was recorded on imaging. For participants with first documented response (CR or PR) and first documented imaging PD, an additional imaging assessment was required at 4 cycles (±7 days) to confirm response and PD. For discontinuation of treatment for reasons other than imaging PD, imaging evaluation may be performed according to the imaging evaluation time cut-off until any of the following events occur: initiation of new anti-tumor therapy, PD, withdrawal of the participant from ICF, and/or death.

20. Participants are required to provide archived or fresh tumor tissue samples that meet the testing requirements during the screening period. In the case of obtaining the ICF of the participant, the surgical resection can be entered at the end of the treatment and the pathological response rate can be evaluated after the procedure.

21. With the participant's ICF, approximately 16ml of peripheral blood can be taken during the screening period, each tumor evaluation period, and at the end of treatment for tumor markers.

22. Termination of treatment refers to confirmation of disease progression or withdrawal from the study, which takes place within ±3 days of the decision to terminate treatment and/or withdraw from the study.

23. Safety follow-up will be conducted at 90±7 days after the last dose.

24. Survival follow-up: telephone visits were taken once every 8 weeks (±7 days) after the last dose.

Abbreviations list

Abbreviation	Full spelling
AE	Adverse event
ALB	Albumin
ALP	Alkaline phosphatase
ALT	Alanine aminotransferase
ANC	Absolute neutrophil count
APTT	Activated partial thromboplastin time
AST	Aspartate transferase
BASO	Basophil cell count
CFDA	China food and drug administration
BUN	Blood urea nitrogen
CK	Creatine kinase
CK-MB	Creatine kinase isoenzyme
CR	Complete response
Cr	Creatinine
CRF	Case report form
CrCl	Creatinine clearance rate
DCR	Disease control rate
DLT	Dose-limiting toxicity
DOR	Duration of response
EC	Ethics committee
EDC	Electronic data capture
ECOG PS	/
EOS	Eosinophil count
FT3	Free thiiodothronine 3
FT4	Free thyroxine 4
FSH	Follicle-stimulating hormone
GCP	Good Clinical Practice

GMP	Good Manufacturing Practices
HCT	Hematocrit value
HGB	Hemoglobin
HRT	Hormone replacement therapy
IB	Investigator’s brochure
ICF	Inform consent form
INR	International normalized ratio
IU	International unit
irAE	Immuno-related adverse event
LDH	Lactic dehydrogenase
LYM	Lymphocyte count
MONO	Monocyte count
MTD	Maximum tolerated dose
Na+	Plasma sodium
Nab	Neutralizing antibody
NSCLC	Non-small cell lung cancer
NYHA	New York Heart Association
ORR	Overall response rate
OS	Overall survival
PBMCs	Peripheral blood mononuclear cells
PD	Progressive disease
PD-1	Programmed death-1
PD-L1	Programmed death-ligand 1
PFS	Progression free survival
PLT	Platelet
PR	Partial response
PT	Prothrombin time
RBC	Red blood cell
Q3W	Every 3 weeks

RECIST	Response Evaluation Criteria In Solid Tumors
RPLS	Reversible posterior leukoencephalopathy syndrome
SD	Stable disease
T3	Triiodothyronine
T4	Thyroxine
TEAE	Treatment emergent adverse event
TCR	T cell receptor
TBIL	Total bilirubin
TP	Total protein
TSH	Thyroid stimulating hormone
TT	Thrombin time
TTR	Time to response
SAE	Serious adverse event
SAP	Statistical analysis plan
UALB	Urinary albumin
UGLU	Urine glucose
UPRO	Urine protein
URBC	Urine red blood cell
WBC	White blood cell
γ-GT	γ-glutamyl transferase

1. Background

Pulmonary nodules refer to a single or multiple nodules with clear boundary, opaque imaging, diameter less than 3cm, surrounded by air-containing lung tissue, without atelectasis, hilar enlargement and pleural effusion. According to whether the pulmonary nodules can completely cover the lung parenchyma under CT, the pulmonary nodules can be divided into solid nodules and subsolid nodules, and the latter can be subdivided into pure ground glass nodules and partial solid nodules. In recent years, with the development of imaging technology and equipment, especially the popularization of multi-slice spiral CT, the detection rate of pulmonary nodules has increased significantly.

Classification based on nodule type and probability of malignancy (very low: <5%; Low-moderate: 5%-65%; High degree: >65%). There are three basic management strategies for patients with pulmonary nodules: (1) surgical treatment; (2) non-surgical biopsy; (3) Continuous CT scanning and close follow-up observation. Pulmonary nodules were classified as single or multiple pulmonary nodules. For multiple pulmonary nodules >1cm in diameter, reexamination 3 months after the first CT examination, if the lesion persists, unless the patient cannot tolerate surgery, non-surgical biopsy or surgical treatment is recommended.

In the process of tumor occurrence and development, tumor cells have many genetic and epigenetic changes compared with normal cells, and theoretically have enough antigens to be recognized by the human immune system, and then trigger an immune response to inhibit tumor growth. In fact, tumors evade the immune system by suppressing the immune response against tumors in a variety of ways. With the deepening understanding of the body's immune system and the rapid development of biotechnology, immunomodulatory therapy has become an important means of cancer treatment, and occupies a more and more important position in the comprehensive treatment system of cancer.

PD-1 (programmed cell death protein-1), a molecule originally thought to be associated with cell death. With the deepening of research, scientists found that PD-1 is not related to programmed cell apoptosis, but has the function of negative regulation

of immunity. PD-1 is an inhibitory receptor mainly expressed on T cells. Under normal physiological conditions, PD-1 can inhibit the activation of T cells and the production of cytokines by binding to its two ligands (PD-L1 / PD-L2), thereby protecting the body from the attack of the autoimmune system. Tumor cells can successfully evade recognition and attack by the body's immune system through the combination of these PD-L1 molecules and PD-1 on T cells. Anti-PD-(L)1 antibody can block this 'tumor immune escape mechanism' and restore the anti-cancer activity of the patients' immune system.

Anti-PD-1 antibody has been successfully used in many types of cancer, including lung cancer, and has become a standard treatment choice for the first and second line. Some advanced patients have achieved durable remission and long-term survival. In recent years, PD-1 inhibitor has also achieved excellent results in earlier neoadjuvant therapy, with a significantly higher response rate than advanced patients. Combined with the principle of immunotherapy, it is generally believed that PD-1 inhibitor is more effective in early tumors. Therefore, this study aimed to explore the efficacy of PD-1 inhibitor on multiple primary lung cancer in the early stage.

Clinical implications of this study: given the remarkable efficacy of immunotherapy in advanced NSCLC and the urgent need to treat unresectable nodules in patients with MPLC, it is promising to investigate the utility of PD-1 inhibitors on early-stage GGO lesions.

2. Study endpoint

2.1 Primary endpoint:

Objective Response Rate (ORR)

2.2 Secondary endpoint:

The safety of sintilimab

3.1 Study design

This is a single center, single-arm, phase II study using the Simon's optimal two-stage design

3.2 Sample size

According to previous studies, the ORR of anti-PD-1 antibody monotherapy for non-small cell lung cancer (NSCLC) was assumed to be 20%, and the ORR of routine observation was not more than 5%. According to the optimal design principle of Simon Phase II trial, α=0.05 (bilateral), β=0.2, the sample size of phase I was calculated to be 10 cases. If no one case was effective, the trial was terminated. If 1 case or more is effective, then enter the second stage, continue to enroll 19 cases, combined with the first stage, a total of 29 cases, according to the 20% of dropping out rate, the study needs to enroll 36 cases.

4. Patients screening

4.1 Inclusion criteria

1) Above 18 years of age;

2) Two or more GGO lesions (pure GGO or GGO-predominant) that cannot be resected simultaneously;

3) There was at least one lesion with a diameter of 1-3cm pathologically confirmed as lung cancer;

4) ECOG PS 0-1 score;

5) The function of vital organs meets the following requirements:

Absolute counting of neutrophil ≥1.5×109/L;

Platelet ≥90×109/L;

Hemoglobin ≥9g/dL;

Thyroid-stimulating hormone (TSH) ≤ULN;

Bilirubin ≤ULN;

ALT and AST≤1.5 ULN;

AKP≤2.5 ULN;

Serum creatinine ≤ 1.5 ULN or creatinine clearance≥60mL/min

6) Women of childbearing age must already be using reliable contraception or have had a pregnancy test (serum or urine) with a negative result within 7 days prior to inclusion and be willing to receive appropriate method of contraception during the

trial and 8 weeks after the last trial drug administration. For males, using appropriate methods of contraception or surgical sterilization are needed during the trial period and 8 weeks after the last course of medication;

7) The participants voluntarily joined the study and signed the informed consents. The participants had good compliance and cooperated with follow-up visits.

4.2 Exclusion criteria

Patients will be excluded if they meet one of below conditions:

1) Non-calcified lesions with a diameter of > 3cm were present

2) Patients with distant metastases were excluded

3) The presence of any active autoimmune disease or a history of autoimmune disease (as follows, but not limited to: autoimmune hepatitis, interstitial pneumonia, uveitis, enteritis, hepatitis, hypophysitis, vasculitis, nephritis, hyperthyroidism, reduced thyroid function; participants who had vitiligo or had complete remission of asthma in childhood could be included without any intervention as adults; Asthma in which participants require medical intervention with bronchodilators is not included);

4) Patients who have used other anti-tumor drugs in clinical study within 4 weeks;

5) Patients with severe allergic reaction to monoclonal antibody;

6) Clinical symptoms or diseases of the heart that are not well controlled, such as: a. NYHA grade 2 or above heart failure; b. Unstable angina pectoris; c. Myocardial infarction within 1 year; d. Clinically significant supraventricular or ventricular arrhythmias requiring treatment or intervention;

7) Participants with congenital or acquired immune deficiency (such as HIV infected persons), or active hepatitis (hepatitis B reference: HBsAg positive, HBV DNA≥2000 IU/ml or copy number ≥104/ml; Hepatitis C reference: HCV antibody positive.

4.3 Exist criteria

1) The participant withdraws informed consent and requires withdrawal;

2) During the enrollment period, participants were required to undergo surgical resection of target lesions;

3) Imaging examination showed the progression of the disease;

4) Those who cannot tolerate toxicity;

5) The participants s lost follow-up or had positive HCG;

6) Other situations in which the investigator deems it necessary to withdraw from the study.

4.4 Termination criteria

1) Finding that there is an unexpected, significant or unacceptable risk to the participants;

2) The investigational drug/trial treatment does not work, or it is pointless to continue the trial;

3) The applicant decides to terminate the study due to reasons such as significant delay in participant inclusion or frequent protocol deviations.

5. Treatment regimen and dosage

200mg sintilimab, intravenous drip, 3 weeks as a treatment cycle, the efficacy was evaluated after 2 courses of treatment. The drug will be discontinued owing to the occurrence of PD, intolerable toxicity, death, withdrawal of informed consent from patients.

6. Dose adjustment and discontinuation

6.1 General guidelines for dose adjustment

- ✓ Reasons for dose adjustment or delay, measures taken and results should be recorded in the patient's medical record and electronic case report form (CRF);
- ✓ If concomitant symptoms are present at baseline, the investigator will determine whether the dose will be adjusted according to the level of change in adverse effects. For example, if a participant has grade 1 lethargy at baseline and grade 2 lethargy during the study treatment, this means that the participant has increased his grade 1 lethargy and the dose should be adjusted according to grade 1 toxicity;
- ✓ If there are several adverse effects of different grades or severity at the same time, dose adjustment will be based on the highest observed grade;

- ✓ If dose adjustment is required only because of laboratory hematological abnormalities, the dose will be adjusted according to the hematological test values before the start of the treatment cycle;
- ✓ Continuation of the current dose without dose adjustment or discontinuation of treatment if the adverse effect is judged by the investigator to be unlikely to develop further serious or life-threatening events. In addition, there will be no dose adjustment or suspension of treatment for anemia (non-hemolytic), which can be relieved by blood transfusion.

6.2 Dose adjustment of sintilimab

No increase or decrease in the dose of sintilimab was allowed, only suspension or termination was allowed.

For the dose adjustment of sintilimab, refer to Table 1; for the infusion reactions related to sintilimab, refer to Table 2.

In addition, investigator-assessed PD according to RECIST v1.1 criteria (unless the participant meets the criteria for continuation of treatment after progression, the drug should be permanently discontinued).

Table 1. Provisions for dose adjustment of sintilimab

Adverse events (AEs)	Severity	Adjustment
**Pneumonia**	Grade 2	Suspension [a]
	Grade 3 or 4	Termination
**Diarrhea**	Grade 2 or 3	Suspension [a]
	Grade 4	Termination
**Dermatitis**	Grade 3	Suspension [a]
	Grade 4	Termination
**Hepatitis**	Grade 2	Suspension [a]
	Grade3 or 4	Termination
**Hypophysitis**	Grade 2	Suspension [b]
	Grade 3 or 4	Termination
**Adrenocortical insufficiency**	Grade 2	Suspension [b]
	Grade 3 or 4	Termination
**Hyperthyroidism**	Grade 3 or 4	Termination
**Diabetes**	Grade 3	Suspension [b]

	Grade 4	Termination
**Renal insufficiency**	Grade 2 or 3	Suspension [a]
	Grade 4	Termination
**Neurotoxicity**	Grade 2	Suspension [a]
	Grade 3 or 4	Termination
**Infusion reaction**	Grade 3 or 4	Termination
**Other AEs**	The other grade 3 AEs appear for the first time	Suspension [a]
	The same grade 3 AEs occur for the second time	Termination
	Grade 3 AEs that cannot fall to grade 0-2/baseline within 7 days or return to grade 0-1/baseline within 14 days	Termination
	Grade 4	Termination [c]

Notes:

a: Resumption of drug administration after symptom improvement to grade 0-1 or baseline level.

b: Hypophysis, adrenocortical insufficiency, and type I diabetes can be readministered when they are adequately controlled and require only physiological hormone replacement therapy.

c: For grade 4 abnormal laboratory findings, discontinuation should be based on accompanying clinical symptoms/signs and on the investigators' judgment.

Table 2. Treatment recommendations for sintilimab-related infusion reactions

CTCAE grade	Dose adjustment	Treatment of toxicity
**Any grade**	-	-Processing according to local clinical practice<br>- Monitor participants for infusion-related reactions (e.g., fever or chills, blush and/or itching, changes in heart rate and blood pressure, dyspnea, chest discomfort, rash, etc.) and allergic reactions (e.g., generalized urticaria, angioedema, asthma, hypotension, tachycardia, etc.)
**Grade 1**	The infusion rate can be reduced by 50% or the infusion can be temporarily interrupted until the infusion reaction is relieved.	For grade 1 or 2:<br>-Administration of acetaminophen and/or antihistamines at investigator discretion according to clinical practice;<br>-Considering prophylaxis prior to administration of
**Grade 2**	The infusion rate can be reduced by	

	50% or temporarily interrupted until the infusion reaction is relieved, and the infusion rate can be adjusted to 50% of the initial rate.	subsequent courses of medication in accordance with clinical practice.
**Grade 3/4**	Termination of medication	For grade 3 or 4:<br>-Manage severe infusion related reactions according to clinical practice (e.g., administration of epinephrine, diphenhydramine, ranitidine, and glucocorticoids)

7. Disables drugs during the study

Participants were prohibited from receiving any of the following treatments for the duration of the study:

- ✓ Systemic chemotherapy and biologic therapies (including antineoplastic agents with immunoregulatory effects, including, but not limited to, interferons, interleukin-2, thymosin, immune cell therapies, etc.);
- ✓ Immunotherapy not specified in this protocol;
- ✓ Study drugs other than those specified in this study, including proprietary Chinese medicines with clear anti-tumor indications;
- ✓ Radiation therapy (Note: Radiation therapy may be approved and permitted by the sponsor for symptomatic isolated lesions or to the brain as long as it is not a lung or target lesion);
- ✓ Live vaccines including but not limited to: measles, mumps, rubella, chickenpox, yellow fever, rabies, BCG, typhoid (oral) vaccine administered within 28 days prior to the first dose of study medication and during study participation. Allow receipt of inactivated vaccine against seasonal influenza by injection; Intranasal live attenuated influenza vaccine (e.g. FluMist®) is not allowed;
- ✓ Corticosteroids, which allow inhaled steroids to be used as part of regular treatment for asthma or chronic obstructive pulmonary disease (COPD). Corticosteroids used to manage potential irAEs are permitted. Physiological doses of corticosteroids are

allowed. Use of prophylactic corticosteroids to avoid allergic reactions (e.g., intravenous contrast) is permitted;

- ✓ At each visit, participants must be asked about any new medications they have received since the previous visit;
- ✓ To reduce the risk of drug interactions, all measures must be taken to limit the number of combinations that are truly necessary;
- ✓ During administration, hepatotoxic drugs (i.e., drugs that are warned of hepatotoxicity in the product label) should be avoided. Investigators are encouraged to review each drug for potential hepatotoxicity by searching the website www.livertox.nih.gov.

8. Study procedure

After signing the informed consent form, each patient will go through three stages to complete the clinical study: screening period, treatment period, and the end of treatment. Before starting the study, patients have to read and sign an informed consent form approved by the Ethics Committee (EC). All study steps need to be performed within the time window specified in the test flow chart. All observation indicators and examination time were not affected by the length of drug withdrawal, and relevant examinations were carried out in each course of treatment according to the regulations.

8.1 Screening Period

Refer to Table 3

Table 3. Procedure of visit during screening period

Phases	Screening period		Notes
	-28-1 Day	-7-1 Day	
**Baseline**			
**ICF**	X		Participants who do not meet the relevant criteria for this study (screening failure) can be re-screened.
**Inclusion/exclusion criteria**	X		
**Demographic/past medical history/past medication**	X		Previous medications include treatments for the initial diagnosis, including chemotherapy, radiotherapy,

			and surgery, and the time of the last previous anti-tumor therapy must be recorded.
**Vital signs**	X		Vital signs include: temperature, pulse rate, respiratory rate, and blood pressure. Refer to protocol 10.1.4
Weight/height	X		
Physical examination	X		Refer to protocol 10.1.2
ECOG score	X		
**Laboratory examination**			
12-leads electrocardiogram	X		Refer to protocol 10.1.3
Blood routine/blood biochemistry/urine routine/stool test		X	Refer to protocol 10.1.1
Coagulation function		X	
Pregnancy tests		X	Serum pregnancy test
Thyroid function	X		Refer to protocol 10.1.7
Myocardial enzyme		X	Refer to protocol 10.1.7
HIV,HBV and HCV	X		Refer to protocol 10.1.7
Ultrasonic cardiogram		X	Refer to protocol 10.1.7
Pulmonary function test	X		Refer to protocol 10.1.7
AE evaluation [17]	X		All AEs from the first dose of study drug to the end of safety follow-up and all SAEs from the signing of ICF to the end of safety follow-up should be recorded in the CRF
**Follow up after treatment**	X		Medications other than solvent associated with treatment AEs should be recorded from 28 days before the first study drug administration to 30 days after the end of treatment and from 30 to 60 days
Survival state			
Subsequent antitumor treatment	X		Refer to protocol 9.2
**Efficacy evaluation**			
Imaging evaluation	X		
**Biomarkers**	X		Sixteen mL of peripheral blood is conserved for biomarker detection
Tissue specimens			
Peripheral Blood			

8.2 Treatment period

Refer to Table 4

Table 4. Procedure of visit during treatment period

Phases	Treatment period			Notes
	Cycle 1 D1	Cycle 2 D1±3	Cycle N D1±3	
**AEs evaluation**	X	X	X	All AEs from the first dose of study drug to the end of safety follow-up and all SAEs from the signing of ICF to the end of safety follow-up should be recorded in the CRF
**Efficacy evaluation**				
**Imaging evaluation**		X	X	Refer to protocol 9.2 and 9.3
**Drug**				
**Sintilimab**	IV，Q3W			
**Biomarkers**				
**Peripheral Blood**		X	X	Sixteen mL of peripheral blood is taken during each tumor evaluation period

8.3 End of Treatment

End of treatment refers to confirmation of PD or withdrawal from the study. An end-of-treatment visit was required ±3 days after the decision to discontinue treatment and/or withdraw from the study (refer to Table 5).

Table 5. Procedure of visit after treatment

Phases	Treatment ending ±3 days	Notes 21days（±7 days）
**Vital signs**	X	Vital signs include: temperature, pulse rate, respiratory rate, and blood pressure. Refer to protocol 10.1.4
**Weight**	X	
**Physical examination**	X	Refer to protocol 10.1.2
**ECOG PS score**	X	
**Laboratory examination**		
**12-leads electrocardiogram**	X	
**Blood routine/blood biochemistry/urine routine/stool examination**	X	Refer to protocol 10.1.1
**Coagulation function**	X	
**Pregnancy tests**	X	Serum pregnancy test
**Thyroid function**	X	
**Myocardial enzyme**	X	
**Ultrasonic cardiogram**	X	
**AEs evaluation**	X	All AEs from the first dose of study drug to the end of safety follow-up and all SAEs from the signing of ICF to the end of safety follow-up should be recorded in the CRF

**Efficacy evaluation**		
**Imaging evaluation**	X	Refer to protocol 9.2
**Biomarkers**		
**Peripheral Blood**	X	Sixteen mL of peripheral blood is taken for tumor markers detection

8.4 Follow-up of safety

Follow-up of safety is performed 90±7 days after the last dose (refer to Table 6).

Table 6. Procedure of follow-up of safety

Phases	Safety follow-up 42±7 days	Notes
**Vital signs**	X	Vital signs include: temperature, pulse rate, respiratory rate, and blood pressure. Refer to protocol 10.1.4
**Weight**	X	
**Physical examination**	X	Refer to protocol 10.1.2
**ECOG PS score**	X	
**Laboratory examination**		
**12-leads electrocardiogram**	X	
**Blood routine/blood biochemistry/urine routine/stool examination**	X	Refer to protocol 10.1.1
**Coagulation function**	X	
**Thyroid function**	X	
**AEs evaluation**	X	All AEs from the first dose of study drug to the end of safety follow-up and all SAEs from the signing of ICF to the end of safety follow-up should be recorded in the CRF
**Visit after treatment**		
**Living state**	X	
**Efficacy evaluation**		
**Imaging evaluation**	X	For discontinuation of treatment for reasons other than imaging PD, imaging evaluation may be performed according to the imaging evaluation time cut-off until any of the following events occur: initiation of new anti-tumor therapy, PD, withdrawal of ICF by the participant, or death.

9. Clinical assessment

9.1 Primary efficacy assessment

Objective response rate (ORR): refers to the proportion of patients whose tumor shrinks by a certain amount of time, including CR and PR cases. Objective tumor response was assessed using the Response Evaluation Criteria in Solid Tumors (RECIST version 1.1). Participants had to have a measurable tumor at baseline, and response was assessed according to RECIST, version 1.1, as complete response (CR), partial response (PR), stable response (SD), or progressive response (PD).

9.2 Secondary efficacy assessment

AEs: Adverse events were recorded from enrollment to 90 days after the end of treatment, and were judged to be related to the treatment drug.

9.3 Imaging scanning

The imaging method used for tumor evaluation at baseline must be consistent with the method used for each subsequent follow-up evaluation, and computed tomography (CT) or magnetic resonance imaging (MRI) scans are recommended. Other examinations are performed depend on the symptoms and signs of each participant.

9.4. Evaluation time points

200mg Sintilimab, intravenous drip, every 3 weeks as a treatment cycle, a total of 4 cycles.

Baseline tumors will be evaluated within 28 days before the first dose, and imaging data obtained before signing informed consent can be used for screening tumor evaluation as long as protocol requirements are met. Imaging evaluation is performed after every 2 cycles of medication until PD was recorded on imaging. For participants with first documented response (CR or PR) and first documented imaging PD, an additional imaging evaluation was performed 3 weeks (±7 days) to confirm response and PD.

Criteria for the participant to PD again:

- ✓ An increase of ≥20% and an absolute increase of at least 5 mm in the sum of target lesion diameters over the lowest value (the smallest sum of diameters, which may occur at baseline or subsequent visits) at 2 consecutive visits, and/or;
- ✓ At the confirmed PD time point, non-target or new lesions have significantly progressed (aggravated so that the total tumor burden has increased enough to stop

treatment even if the target has CR, PR, or SD) relative to the first time point at which non-target progression or new lesions were detected, and/or;

- ✓ Additional new lesions at confirmed PD time points relative to the first time point at which new lesions were detected.

If the PD was not confirmed, medication was continued and imaging assessment was performed until re-PD. Treatment should be terminated for participants with re-PD. For discontinuation of treatment for reasons other than imaging PD, imaging evaluation may be performed according to the imaging evaluation time cut-off until any of the following events occur: initiation of new anti-tumor therapy, PD, withdrawal of ICF by the participant, or death.

10. Safety Evaluation

10.1 Safety indicators

10.1.1 Routine laboratory safety indicators

Table 8. Routine laboratory safety assessment

**Blood routine examination**	RBC, HGB, HCT, WBC, PLT, LYM, ANC, MONO, EOS and BASO
**Coagulation function**	TT, PT, APTT, and INR
**Blood biochemistry**	TBIL[a], ALT, AST, γ-GT, ALP, ALB, TP, LDH, BUN, Cr, Na, K, Cl, Mg, Ca, P, lipase, amylase and FBG
**Routine urine test**	PH, UALB, UPRO[b], URBC and UGLU
**Stool examination**	Occult blood

Notes:

a. If TBIL≥2×ULN (and there is no evidence of Gilbert syndrome), then measure direct and indirect bilirubin separately.

b. White blood cells should be examined by microscopy (if appropriate) and red blood cells by high magnification.

10.1.2 Physical examination

A complete physical examination includes: general condition, respiratory, cardiovascular, abdominal, skin, head and neck (including ears, eyes, nose and throat), lymph nodes, thyroid, musculoskeletal (including spine and limbs), genital/anal, and neurological evaluation.

10.1.3 12-lead ECG

The resting 12-lead ECG will be analyzed at the local laboratory according to the visit schedule.

A 12-lead ECG is performed in each case after the participant rested in the recumbent position for at least 5 min. All 12-lead ECGs should be recorded while the participant is resting in the supine position. Further ECG will be performed when there is a clinical need such as in the event of a heart-related AE. The investigators completed the ECG assessment on the day of the examination, and the assessment results were recorded on the electrocardiogram. The same assessment method should be used throughout the study period.

The ECG will be recorded at a speed of 25 mm/sec. The investigators should evaluate all ECGs according to the clinically significant abnormality/no clinically significant abnormality category. If it is an abnormal result of clinical significance, the investigator should record the result as an AE in the CRF.

10.1.4 Vital signs

Vital signs will be performed as described in the study visit schedule. Vital signs include body temperature, pulse rate, respiratory rate, and blood pressure. Additional vital sign assessment monitoring may be performed according to standard clinical practice or at the investigator's discretion based on clinical need. In the presence of AEs/SAEs, additional vital sign recording values may be collected on the CRF, if applicable. The date and time of acquisition and measurement will be recorded in the appropriate section of the CRF.

Pulse and blood pressure

Pulse and blood pressure were collected during the screening period and before the planned administration of sintilimab infusion.

Blood pressure monitoring: During each blood pressure measurement, smoking and coffee drinking should be prohibited within 30 minutes before the measurement, and at least 10 minutes of quiet rest should be taken. During the measurement, the elbow should be placed in the sitting position at the same level as the heart, and each blood pressure measurement should be taken on the same side.

Blood pressure was measured by the investigator during the screening period and before each planned sintilimab infusion. During the study, blood pressure monitoring was completed by the participants themselves and recorded in the participants' diary card. Blood pressure was measured at least 3 times a week in the first 2 cycles, and if blood pressure was abnormal, it was tracked every day. If the blood pressure was normal, the blood pressure was checked twice a week after the third cycle.

Body temperature and respiration

Body temperature and respiration were collected during the screening period and before the planned daily infusion of sintilimab.

10.1.5 Height and weight

Height measurements are performed only during the screening period, and weight measurements are required during the screening period, before each visit to sintilimab, at the end of treatment, and at the safety follow-up.

10.1.6 Pregnancy reaction

Pregnancy tests are performed for serum human chorionic gonadotropin (hCG) samples within 7 days before the first dose of the study drug. If the result is positive, the participant is not eligible for inclusion/must stop participating in the study. Pregnancy is suspected to have occurred during the study and should be reviewed.

10.1.7 Other safety checks

- ✓ Hepatitis B five: HBsAg, HBsAb, HBcAb, HBeAg, HBeAb
- ✓ HIV antibodies, HCV antibodies
- ✓ Thyroid function: T3, T4, TSH, FT3, and FT4

- ✓ Myocardial enzyme profile: LDH, AST, CK, CK-MB, and ALT
- ✓ Pituitary-adrenal axis examination: including CRH, plasma ACTH, and adrenocortical hormone measurements. Adrenocortical hormone assays included serum cortisol, UFC, 17-KS, and 17-KGS
- ✓ Echocardiography
- ✓ Pulmonary function test: VT, BE, MV, ERV, IC, VCmax, FVC, FEV1, FEV1/FVC, PEF, MEF75, MEF50, MEF25, MVV, TLC, RV/TLC, BR, DLCO SB, Raw eff and sGaw eff

10.2 Observation of AEs

Adverse Events (AEs) definition: An AE refers to any adverse event that occurs in a participant or clinical study participant after receiving a drug or treatment regimen, but is not necessarily causally related to the treatment.

An AE may be any unpleasant sign (including abnormal laboratory test results), symptom or illness that is temporally associated with or unrelated to the use of the medical product, whether or not it is thought to be related to the medical product.

According to the regulations, events that occurred during the pre-and post-treatment phase were also considered as AE. Therefore, safety monitoring AE or SAE should be reported from the time a participant enters the trial (after signing an informed consent form) until the end-of-trial visit.

10.3 AE Classification

AE was graded as 0-5 according to NCI Common Acute and Subacute Toxicity Grading Criteria (NCI-CTCAE 5.0). AEs not listed in the NCI toxicity classification criteria can be determined according to the following criteria:

Grade I: mild, no clinical symptoms or clinical symptoms of the youth committee; Only clinical or laboratory abnormalities; No treatment is required; Does not interfere with normal daily activities;

Grade II: moderate, requiring minimal, topical, or noninvasive treatment; Interfere with normal daily activities (cooking, shopping, talking on the phone, counting money, etc.)

Grade III: severe illness or medical serious symptoms single temporarily not life-threatening; Resulting in hospitalization or prolonged hospitalization; Cause disability;

Unable to work or perform normal daily activities (bathing, dressing, undressing, eating, going to the bathroom, taking medication, etc.), not bedridden;

Grade IV: life-threatening, requiring urgent treatment;

Grade V: Death due to adverse events.

10.4 AE Records

The name, severity, occurrence time, duration, treatment measures, and outcome of various AE occurred during the trial were recorded in detail, and truthfully filled in the Case Report Form (CRF). Abnormal laboratory test data were recorded on the CRF form, and the test was repeated at least once a week until recovery to normal or the end of the study. Adverse events that occur within 30 days of the last dose should be reported and recorded.

10.5 Judgment of the relationship between AE and test drugs

Possible associations between AE and test drugs were assessed on a five-point scale of "definitely related, very likely related, probably related, probably not related, and not related". The first three levels were judged to be related to the test drug, and the total number of participants used to evaluate the safety was used as the denominator in the calculation of the incidence of adverse reactions.

10.6 Severe adverse events (SAEs)

1) Severe adverse events (SAEs) refers to the occurrence of medical events in the process of clinical research, such as requiring hospitalization or prolonged hospitalization, disability, affecting work ability, endangering life or death, and causing congenital malformations. These include the following unexpected medical events: events leading to death; Life-threatening events (defined as when the participant is in danger of death during the event); Events requiring hospitalization or prolonged hospitalization; Events that can result in permanent or severe disability/disability; Carcinogenic or teratogenic.

2) Drug exposure during pregnancy/lactation. In principle, pregnancy and lactation are inclusion and exclusion criteria. If pregnancy occurs during the study, the patient should withdraw from the study immediately, notify the investigator immediately, and follow up the patient throughout the pregnancy and postpartum. Even if both mother and child

are completely normal without any adverse events, the consequences should be documented. Even if the pregnancy is not an SAE, it should be reported using the SAE report form.

3) Disease progression, including signs and symptoms of progression, should not be reported as a serious adverse event, except for death from disease progression during the trial or safety reporting period. Hospitalization for symptoms and signs of disease progression should not be reported as a serious adverse event. During the trial or safety reporting period, if the final outcome of the cancer is death, then the event leading to death must be reported as a serious adverse event.

4) Other antineoplastic therapy: If the participant starts other antineoplastic therapy, the reporting period for adverse events that are not death is up to the start of the new antineoplastic therapy. Deaths that occurred within the reporting period for SAEs after the end of the study treatment had to be reported regardless of whether the patient was receiving additional treatment.

5) Hospitalization: Adverse events leading to hospitalization or prolonged hospitalization in clinical studies should be considered as SAEs. Any initial admission to a medical facility, even if less than 24 hours, met this criterion. Hospitalization does not include: rehabilitation facility, nursing home, routine emergency room admission, same-day surgery (e.g., outpatient/same-day/ambulatory surgery), hospitalization unrelated to worsening of adverse events, or prolonged hospital stay that is not itself a serious adverse event, e.g., admission for a pre-existing medical condition with no new adverse event or exacerbation of an existing medical condition (e.g. To check for laboratory abnormalities that have persisted since before the test); Hospitalization for administrative reasons (e.g., annual routine physical examination); Protocol-mandated hospitalization during the clinical study (e.g., as required by the protocol); Elective hospitalizations not associated with worsening adverse events (e.g., elective cosmetic surgery); Scheduled treatments or surgical procedures should be documented throughout the trial protocol and/or in the baseline data of individual participants; Admission for blood product use alone. Diagnostic or therapeutic invasive (e.g., surgery), non-invasive procedures should not be reported as adverse events. However,

it should be reported when the condition that led to the procedure meets the definition of an adverse event; for example, acute appendicitis that developed during the reporting period should be reported as an adverse event, and the resulting appendectomy should be recorded as the treatment for that adverse event.

6) Drug overdose: Drug overdose refers to the participant's addition of the test drug within 24 hours (the specific time is adjusted according to the specific protocol), and the dose is higher than the dose prescribed by the investigator's order. All trial drug overdoses, regardless of whether they were associated with adverse/severe adverse events, should be reported as SAEs.

10.7 SAE reporting procedures

SAEs are reported from the time the participant gave informed consent until 30 calendar days after the last use of the study drug. During the trial, any SAE must be reported to the clinical supervisor and the principal investigator within 24 hours. At the same time, the "New Drug Clinical Study Severe Adverse Events (SAE) Report Form" must be completed, signed and dated. And immediately report by fax to the sponsor unit, the group leader unit, the ethics committee of the research unit, the State Food and Drug Administration (CFDA) and the food and drug Administration of the region (province or city) where the researcher is located.

SAEs must be reported to the sponsor within 24 hours of the discontinuation of the study. Information on all SAEs should be recorded in the SAEs table. SAEs that occur between the continuation of the drug and within 30 days after the last dose must be reported. SAEs that occurred 30 days after the last dose were generally not reported unless they were suspected to be related to the study drug.

SAEs should be documented in detail in terms of symptoms, severity, time of occurrence, time of management, measures taken, time and mode of follow-up, and outcome. If the investigator believes that a serious adverse event is not related to the trial drug but is potentially related to a study condition (e.g., discontinuation of the original treatment or coexisting conditions during the trial), this relationship should be detailed in the description section of the SAEs page of the medical record report form. If there was a change in the intensity of an ongoing serious adverse event or its

relationship to the trial drug, a follow-up report of a serious adverse event should be sent to the sponsor immediately. All SAEs should be followed up until recovery or stabilization.

11. Data collection and management

11.1 Data entry and modification

Data entry and management are handled by an independent data administrator. If there are any questions in the case report form, the data manager will write them into the Question Answering Form (DRQ), and send them to the researcher through the clinical monitor. The researcher should answer and return as soon as possible. The data manager will modify, confirm and enter the data according to the answers of the researcher, and send the DRQ again if necessary.

11.2. Data locking

After data review and confirmation that the established database was correct, the data were locked by the principal investigator and statistical analyst. The locked data file is not modified anymore.

11.3. Statistical analyses

The results of this test are mainly described by statistical methods. The mean, standard deviation, median, maximum and minimum values were listed for measurement data, and the frequency (constituent ratio), rate and confidence interval were listed for count data and grade data.

All statistical analyses will be calculated programmatically using SPSS Statistics (version 23.0, IBM). All statistical tests were two-sided; a p-value of 0.05 or less was considered statistically significant, and 95% confidence was used for the confidence interval.

Basic characteristics of patients: the mean, standard deviation, median, maximum and minimum values of quantitative data such as age, height and weight were calculated, and the frequency and percentage of qualitative data such as gender and ECOG score were listed.

Efficacy analysis: ORR was the primary efficacy index. Objective response rate (ORR=CR+PR) and 95% confidence interval (CI) were calculated.
Safety evaluation: Descriptive statistical analysis is used to describe the adverse events that occurred in this trial. Laboratory test results describe normal conditions before the test but abnormal conditions after treatment and the relationship between abnormal changes and the test drug when they occur. The mean, standard deviation, median, minimum and maximum values of vital signs and laboratory indicators before and after medication were calculated, and paired t-test was used for comparison before and after medication.

12. Ethical Considerations

This study is conducted in strict compliance with the ethical guidelines for human medical research of the Declaration of Helsinki and the relevant Chinese clinical research regulations. Before starting, the protocol study and the informed consent form of the participants were reviewed and approved by the PI unit and the ethics committee before implementation.
During the study, all operations were strictly guaranteed to be carried out in accordance with the approved protocol. Any modification of the study protocol should be submitted to the ethics committee for approval again, and serious adverse events should be reported to the ethics committee of hospital.
Before being enrolled in this study, each participant shall provide a complete, comprehensive and truthful introduction to the patients or their designated representatives about the objectives, procedures, possible risks, benefits and relevant rights and interests of this study in written form, and ensure that each participant voluntarily participates in this study and signs a written informed consent form, so that the rights of the participants are fully protected. All data of the participants are strictly confidential.
